# Temperature- and Pressure-Induced Ligand Anisotropy Drives Structural Reorganization of Dendronized Gold Nanoparticle Monolayers

**DOI:** 10.1021/jacs.5c22437

**Published:** 2026-05-01

**Authors:** Rina Sato, Joshua Reed, Emanuel Schneck, Kiyoshi Kanie

**Affiliations:** † Institute of Multidisciplinary Research for Advanced Materials, 13101 Tohoku University, 2-1-1 Katahira, Aoba-ku, Sendai, Miyagi 980-8577, Japan; ‡ Institute for Condensed Matter Physics, 26536 Technische Universität Darmstadt, Hochschulstrasse 8, 64289 Darmstadt, Germany; § International Center for Synchrotron Radiation Innovation Smart, 13101 Tohoku University, 2-1-1 Katahira, Aoba-ku, Sendai, Miyagi 980-8577, Japan

## Abstract

Self-assembly at the air/water interface provides a versatile platform for organizing organic ligand-functionalized inorganic nanoparticles (NPs) into two-dimensional monolayers. However, how ligand behavior under interfacial confinement governs collective structural organization of NP assemblies remains poorly understood. Here, we demonstrate that ligand redistribution on Au NPs induces emergent NP shape anisotropy, which in turn drives directional reorganization of interfacial monolayers. A monolayer of Au NPs dual-functionalized with a liquid-crystalline dendron and dodecanethiol reorganizes from island-like arrays to network-like structures upon heating. X-ray reflectometry and grazing-incidence small-angle X-ray scattering further reveal correlated variations in out-of-plane ligand-shell thickness and in-plane lattice constants. Integrating these X-ray results with local structural insights from electron microscopy clarifies that adaptive redistribution of the two coexisting ligands on the NP surface was the key factor that changes the NP shape anisotropy. This ligand-driven anisotropy directly induced directional anisotropy of the macroscopic monolayer structure. Such dynamic ligand redistribution is enabled by a precisely engineered NP surface, dual-functionalized with liquid-crystalline dendrons and simple alkanethiols. Altogether, this work establishes a strategy for designing thermoresponsive NP monolayers with tunable topology at liquid interfaces and highlights how interfacial confinement fundamentally alters ligand-mediated assembly behavior.

## Graphical abstract

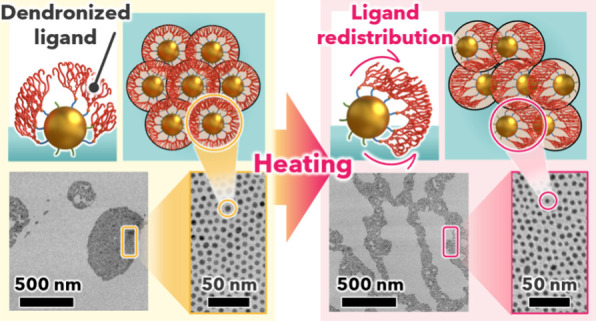

## 1 Introduction

Assembling inorganic nanoparticles (NPs) into one-, two-, or three-dimensional architectures has attracted significant attention owing to the emergence of unique properties arising from interparticle interactions.^1–4^ Among the various strategies, bottom-up self-assembly has been established as a powerful approach, where NPs act as fundamental building blocks.^5–7^ However, bare inorganic NPs rarely undergo spontaneous self-organization into ordered structures and instead tend to aggregate under conditions of reduced interparticle repulsion in order to decrease the surface energy. To overcome this limitation, their surfaces are often covalently functionalized with organic ligands that provide self-assembling capabilities. Such ligands promote uniform NP arrangements through ligand–ligand interactions, stabilizing interparticle gaps. Moreover, incorporating ligands responsive to external stimuli enables structural rearrangements in response to factors such as pH,^1,8^ light,^9,10^ temperature.^11,12^ These dynamic reconfigurations are of great interest, as changes in structural order can modulate interparticle coupling and, consequently, the material’s overall functionality.

Liquid-crystalline (LC) molecules are particularly promising ligands for imparting LC properties to inorganic NPs, enabling dynamic, stimulus-responsive control over NP arrangements.^10,13–15^ In this work, gold nanoparticles (Au NPs) functionalized with dendritic LC molecules and dodecanethiols were spread at the air/water interface, where their two-dimensional (2D) arrangement was thermally modulated via the dendrons’ intrinsic responsiveness. The LC dendron used here (structural formula shown in Figure S1) was specifically designed to undergo phase transitions upon heating, altering its molecular conformation and thereby reorganizing the NP assembly. In this system, the inorganic NPs were first cofunctionalized with dodecanethiol and 16-mercaptohexadecanoic acid (MHA), and the LC dendrons were subsequently connected to the end of MHA. Since the dendron molecules can only be attached via amide bonding to the terminal carboxy group of MHA, this mixed-ligand approach enabled precise control over the surface modification density of the LC dendrons. Previous studies have demonstrated that dendron- and dodecanethiol-dual-functionalized inorganic NPs—including Au NPs,^16^ CdS quantum dot,^17^ and magnetic FePt NPs^18^—with an appropriate surface modification density of dendron formed dendron-mediated self-assembled structures, and that their three-dimensional (3D) superlattices in the dry state can be thermally reconfigured. However, reported phase transitions typically require high temperatures (>100 °C). While such transitions are expected to be able to switch NP properties through structural rearrangement, the high temperature requirement limits practical applicability. Here, we hypothesize that the air/water interface can enhance ligand flexibility, enabling thermal responsiveness at substantially lower temperatures.

This study is motivated by the broader goal of creating thermoresponsive 2D metamaterials through NP film formation at the air/water interface using hybrid NPs composed of inorganic cores and functional organic shells. The assembly of 2D NP arrays at the air/water interface has been widely studied, particularly for NPs modified with hydrophobic or amphiphilic ligands.^19–21^ In this approach, NPs dispersed in an organic solvent are deposited onto a water surface; subsequent solvent evaporation drives the spontaneous formation of a monolayer film immobilized at the interface, known as Langmuir film. Thus, Langmuir films at the air/water interface have been extensively characterized by X-ray techniques to elucidate interfacial molecular and NP structures. For example, studies of lipid monolayers and bilayers have yielded key insights into the orientation and organization of biomolecular assemblies, which are fundamental to biophysics and biochemistry.^22,23^ Similarly, given the active interest in Au NPs functionalized with organic ligands for biomedical and plasmonic applications, it is important to clarify their interfacial behavior. A comprehensive understanding of temperature-induced rearrangements in ligand-functionalized NP films will provide design guidelines for interfacial materials capable of dynamically switching their structure and function.

Despite sustained interest in thermoresponsive materials, real-time structural behavior at liquid interfaces has remained largely unexplored. For instance, poly­(*N*-isopropylacrylamide) (PNIPAM)-functionalized NPs have been investigated for their thermoresponsive behavior at the air/water interface;^24–26^ however, these studies mainly reported the reorganization of the PNIPAM chains themselves, without achieving controlled rearrangement of the NP assemblies at the interface.

Here, we present a thermoresponsive NP liquid film that dynamically reorganizes its 2D arrangement in response to both water temperature and lateral compression through adaptive rearrangement of LC dendron and dodecanethiol ligands on the NP surface. The thermoresponsive behavior of dendronized Au NPs was systematically investigated at the air/water interface using complementary in-plane and out-of-plane X-ray scattering techniques, providing an integrated view of the reorganization mechanism. Furthermore, the combination of these X-ray approaches enabled us to resolve not only the conformational change and the adaptive rearrangement of the ligands on the NP surface under compression and heating, but also how these conformational behaviors of ligands directly drive the collective rearrangement of the NP self-assembled structures at the interface. To our knowledge, this is the first demonstration of adaptive reorganization of self-assembled 2D NP structures at the air/water interface that is explicitly governed by dynamic rearrangement of two kinds of organic ligands—LC dendrons and dodecanethiols—on the NP surface.

## 2 Results and Discussion

A schematic overview of this work is shown in Figure 1. For comparison with the behavior of Au NPs functionalized with both dendron and dodecanethiol (Au-Dend), Au NPs modified with only dodecanethiol (Au-DT), as with nearly the same Au-core diameter as Au-Dend were employed (Figure 1a). Au-DT, modified with only rigid linear alkyl ligands without LC functionality, serves as a reference system. These two types of NPs will be discussed with regard to the same characterization techniques to emphasize the unique thermoresponsiveness of Au-Dend.

**Figure 1:**
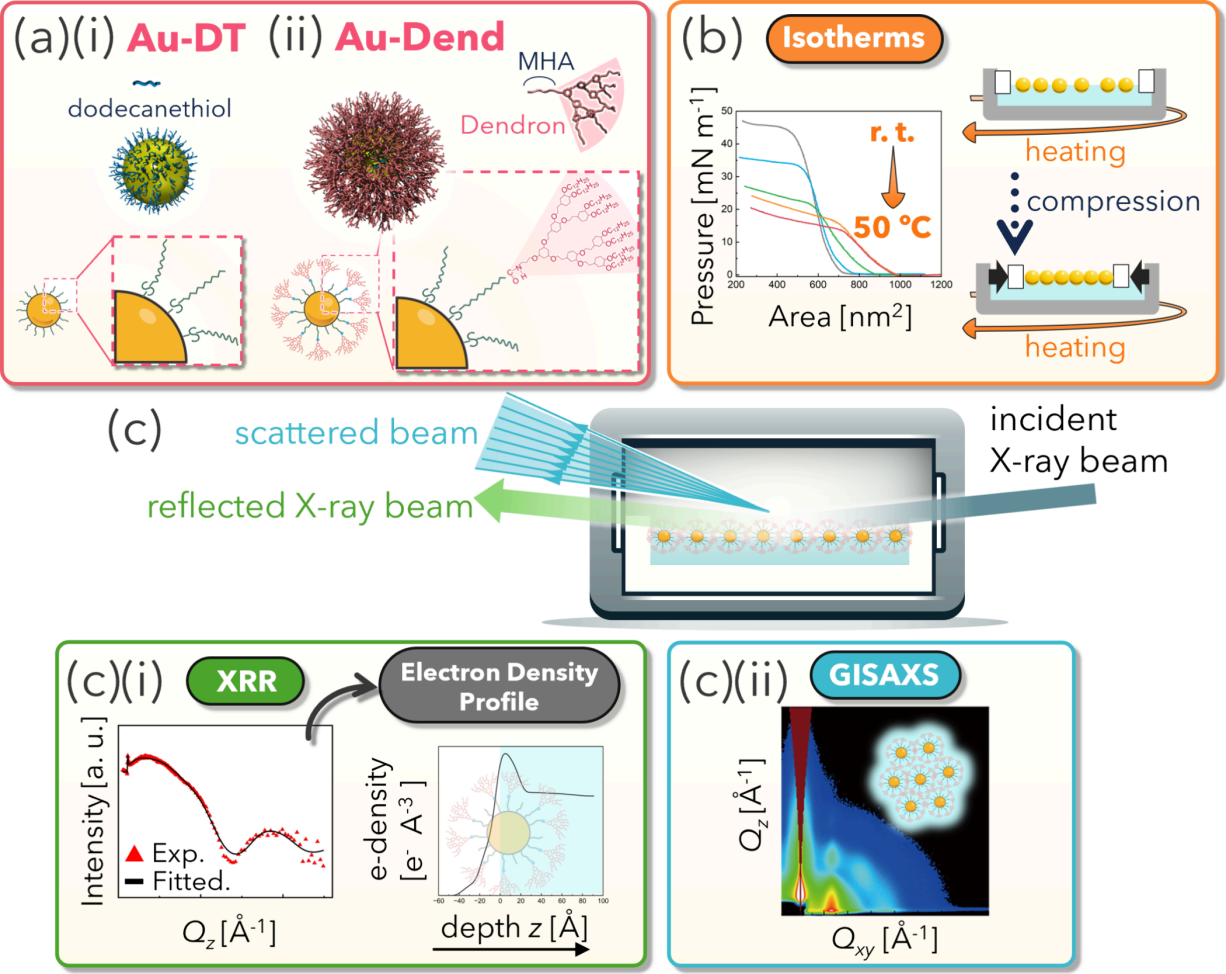
Schematic overview of various techniques for analyzing NP monolayers at the air/water interface. (a) Illustrations of Au NPs (i) modified with dodecanethiol (Au-DT) or (ii) cofunctionalized with dodecanethiol and dendron (Au-Dend). Insets depict enlarged images of the ligand shells. (b) Temperature-dependent surface pressure–area (π–*A*) isotherms of NP monolayers measured in a Langmuir trough while varying the subphase temperature from room temperature to ≲50 °C. Sketches to the right in (b) illustrate the compression and heating sequence. (c) Schematic of surface-sensitive X-ray techniques. (i) X-ray reflectivity (XRR): the specularly reflected beam (green arrow) is collected with a 1D detector to reconstruct the electron density profile normal to the interface, providing out-of-plane structural information on the NP monolayer. (ii) Grazing-incidence small-angle X-ray scattering (GISAXS): scattered beams (blue arrows) collected with a 2D detector yield in-plane ordering information (e. g., hexagonal arrangement shown in the top right).

To control the surface ligand ratio of Au-Dend, Au NPs were cofunctionalized with MHA and dodecanethiol in a 3:2 molar ratio. Previous studies have shown that introducing an appropriate fraction of dendrons is essential for Au NPs to exhibit dendron-derived liquid crystallinity, and the 3:2 modification ratio was identified as suitable for generating a regular structure associated with LC phase transition.^16^ Comprehensive analyses of the interfacial behavior of these NPs were carried out through X-ray measurements and temperature-dependent surface pressure–area (π–*A*) isotherms (Figure 1b). The combination of isotherms and direct observation using transmission electron microscopy (TEM) provides information about the evolution of film flexibility as the thermoresponsive transition of NP assembly. In addition, surface-sensitive X-ray techniques were carried out to probe structural changes in dendrons and NP monolayers (Figure 1c). X-ray reflectivity (XRR) (Figure 1c­(i)) and grazing-incidence small-angle X-ray scattering (GISAXS) (Figure 1c­(ii)) techniques are powerful tools to characterize the interfacial orientation/arrangement along the out-of-plane and in-plane directions, respectively.^27^ In the final section, we discuss the ligand-conformation-induced reorganization of Au-Dend monolayers by integrating insights obtained from both X-ray methods.

### 2.1 Thermoresponsive NP Assembly at the Air/Water Interface

The TEM images of as-synthesized Au-Dend and Au-DT NPs are shown in Figure S2. The average core diameters were determined to be 6.5 ± 0.7 and 6.2 ± 0.7 nm, respectively, indicating that the difference between the two types of NPs lies primarily in the surface ligands. Notably, the interparticle center-to-center distance of Au-Dend (11.9 ± 0.7 nm) was significantly larger than that of Au-DT (7.8 ± 0.2 nm), reflecting the effect of the bulky, branched dendron ligands. The ligand layer thickness was estimated by subtracting the core diameter from the center-to-center distance, yielding values of 5.4 and 1.6 nm for Au-Dend and Au-DT, respectively. The thickness for Au-DT is consistent with the expected length of a fully extended dodecanethiol chain, estimated by (*L* = 0.12­(*n* + 1)) [nm], where *n* is the number of carbon atoms (1.56 nm for *n* = 12). This agreement suggests that the dodecanethiol ligands are radially extended from the NP surface and interdigitated. In contrast, the thicker and softer dendron ligands in Au-Dend lead to reduced uniformity in hexagonal packing.

Figure 2 summarizes the temperature- and pressure-dependent TEM observations of Au-Dend and Au-DT monolayers at the air/water interface. As shown in Figure 2a,b, both types of NPs formed hexagonally ordered assemblies at 20.0 °C and 0 mN m^–1^, and these hexagonal arrangements were maintained upon compression and with increasing water temperature (see, Figures S3 and S4 for enlarged images of Figure 2c,d). However, the large-scale arrangement of Au-Dend exhibited dynamic structural transformations, indicative of thermoresponsive behavior. At 20.0 °C, the typical Langmuir monolayer formation process was observed for Au-Dend (Figure 2c):^28,29^ immediately after spreading, small NP islands formed randomly on the water surface subsequently coalesced into larger domains upon compression. As temperature increased, the assemblies evolved from discrete islands to elongated 1D chain-like structures at 27.9 °C and further into 2D networks above 34.0 °C, with substantial void areas. At 27.9 °C, compression led to the formation of larger islands similar to those at room temperature (rt), while at 34.0 °C and above, island domains were observed at the network junctions and gradually transformed into “island network” structures. In contrast, Au-DT showed a monotonic increase in particle density with compression at both 19.3 and 34.9 °C, without significant morphological rearrangements. Notably, Au-DT began to spontaneously fuse with neighboring particles already at 34.9 °C (enlarged images are shown in Figure S5a,b). Moreover, we confirmed that this fusion became more pronounced at 50 °C in the dry state, where Au-DT no longer maintained a spherical NP shape (Figure S5c). Thus, at elevated temperatures, Au-DT transitions from ligand-mediated interfacial self-assembly to a distinct process dominated by particle coalescence. This behavior contrasts with that of Au-Dend and supports that dendron functionalization enables a more flexible and thermally responsive structural adaptation that cannot be achieved with simple alkyl chains. These results highlight the distinct thermoresponsive and structurally flexible nature of the Au-Dend monolayer compared to the more rigid Au-DT monolayer.

**Figure 2:**
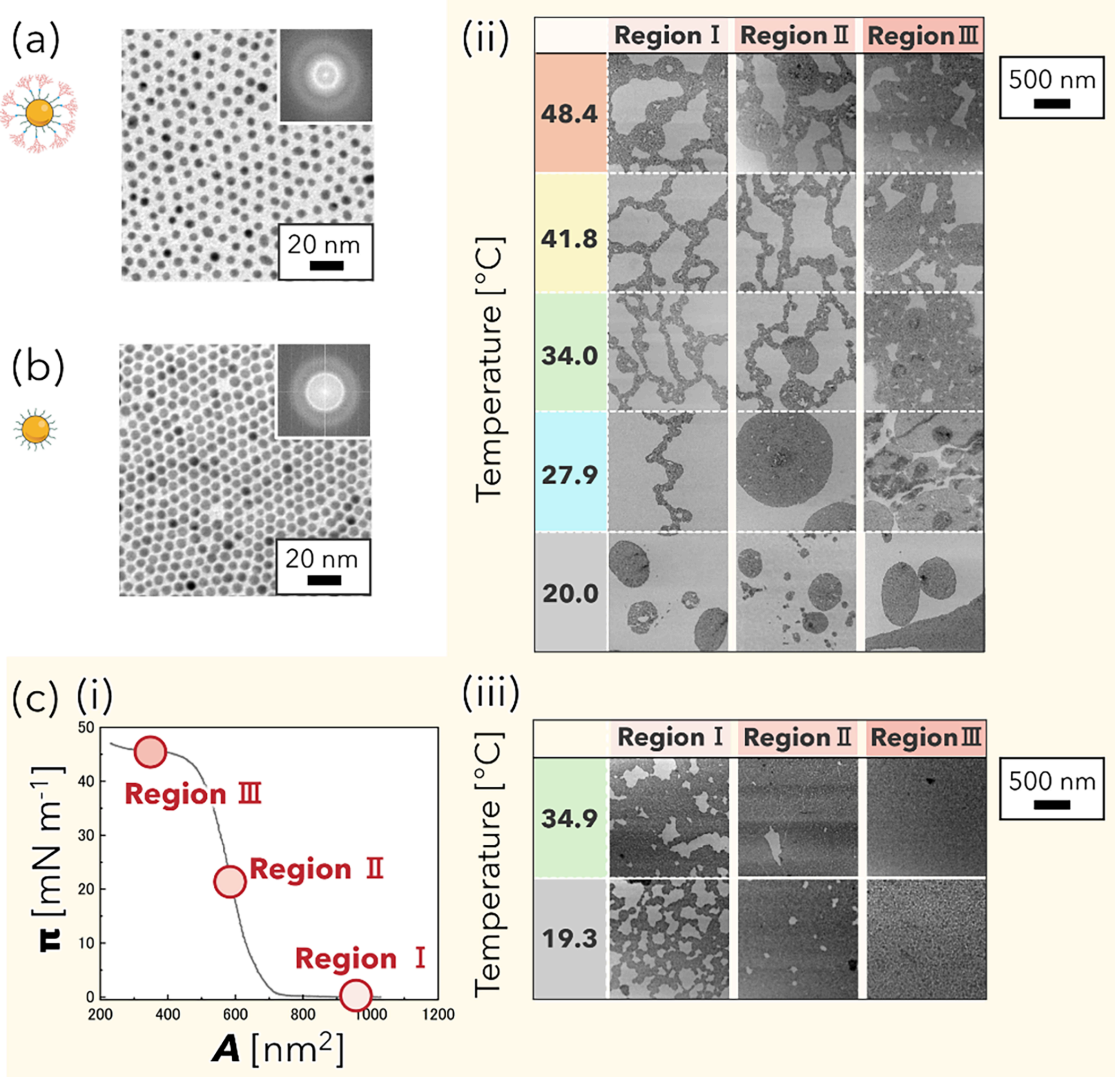
TEM images of (a, c­(ii)) Au-Dend and (b, c­(iii)) Au-DT monolayers at the air/water interface under various surface pressures and temperatures. (i) Representative π–*A* isotherm of Au-Dend at rt, show three regions in (ii) and (iii); immediately after annealing, during the initial increase in surface pressure, and after the transition point (as defined in Section 2.2), respectively. Regions I, II, and III in (c) (ii, iii) at the other temperatures correspond to the same compression stages in the π–*A* isotherms of Au-Dend and Au-DT in Figure 3a,b, respectively. (a, b) Enlarged images of each NP type at 20.0 °C and 0 mN m^–1^. The insets show the corresponding fast Fourier transform (FFT) patterns, revealing hexagonal symmetry. Schematics to the left of the TEM images in (a) and (b) illustrate each type of Au NP.

### 2.2 Surface Pressure–Area Isotherms and Compressibility

Figure 3a,b shows the surface pressure–area (π–*A*) isotherms of Au-Dend and Au-DT monolayers at various water temperatures. Au-DT exhibited a steep rise in surface pressure upon compression at all temperatures, consistent with the formation of typical solid-like monolayers. In contrast, Au-Dend displayed more gradual increases, typically seen in the case of polymer-grafted NP systems or spherically self-organized dendron molecules, where 2D structural rearrangements are tolerant.^24,26,30^ The π–*A* curve obtained during the second compression at rt closely resembled that of the first cycle. This observation suggests a degree of reversibility in the structural response of the Au-Dend monolayer under lateral compression within this pressure range. Remarkably, systematic shifts were observed in both the lift-off points and transition points of Au-Dend monolayer as the temperature increased (Figure 3c). The lift-off point was defined as the area per particle at which the π–*A* curve began to rise, whereas the transition point was defined as the surface pressure corresponding to the intersection of the tangent lines drawn before and after the inflection in each isotherm (see Figure S7). Although the isotherms of Au-DT exhibit minor shape variations at certain temperatures, these differences are attributed to nonequilibrium effects caused by the initial NP distribution—namely, the formation of discrete NP islands rather than uniform surface coverage—as observed in the TEM images in Figure 2d. Unlike amphiphilic molecules that typically form continuous monolayers, NP islands coalesce upon compression, leading to local variations in pressure response. Even though structural reorganization behavior at higher temperature would have been interesting to explore, measurements at higher nominal temperatures were not performed because of the potential influence of evaporation-induced artifacts.

**Figure 3:**
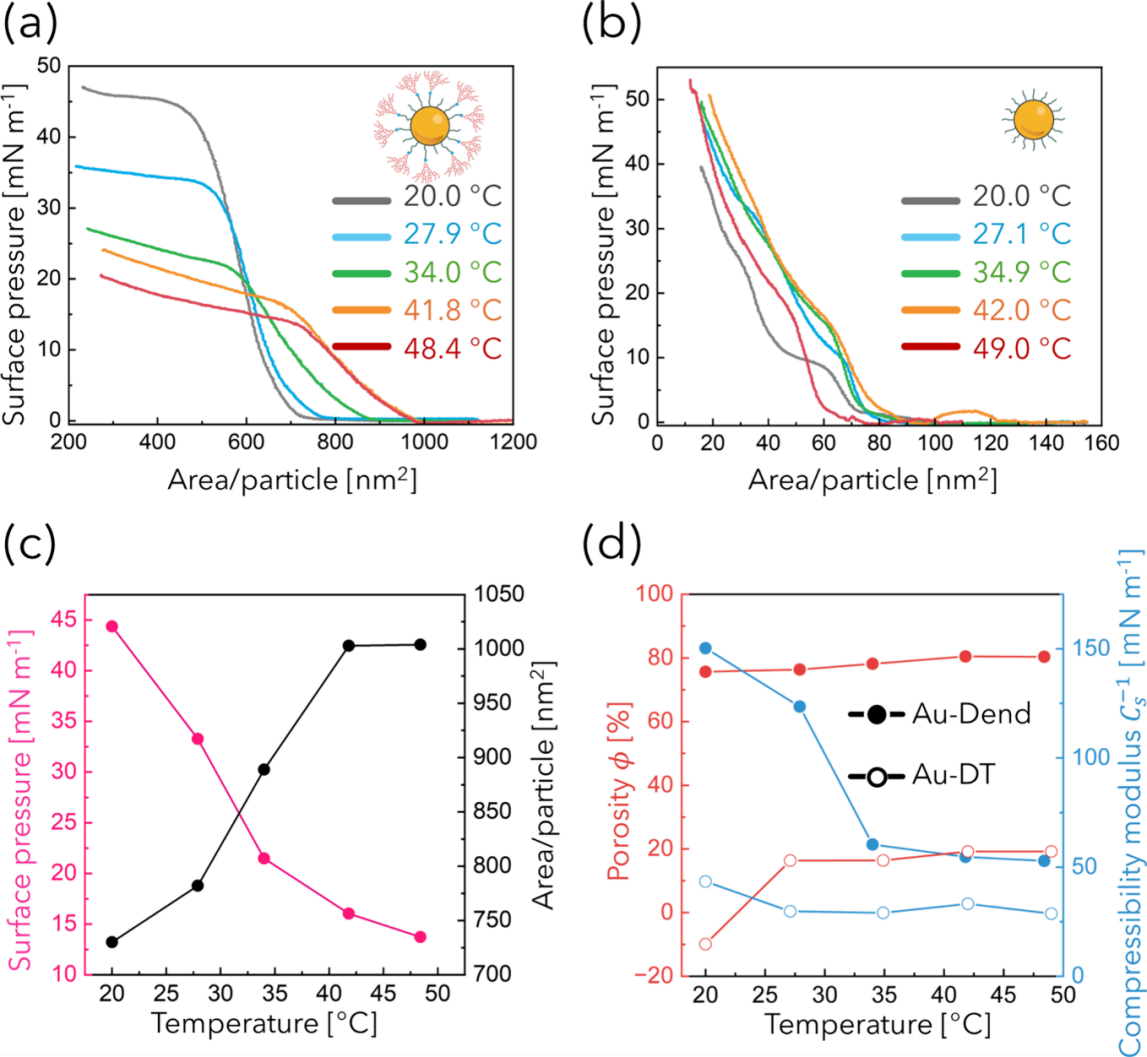
Temperature-dependent surface pressure–area (π–*A*) isotherms of (a) Au-Dend and (b) Au-DT monolayers measured over a nominal temperature range from room temperature (20.0 °C) to ≲50.0 °C. (c) Surface pressure at the transition point (pink) and area/particle at the lift-off point (black) for Au-Dend, extracted from the isotherms in (a), plotted as a function of temperature. (d) Porosity (red) and compressibility modulus (blue) of Au-Dend (filled circles) and Au-DT (open circles), calculated from the isotherms at each temperature.

The features observed in the Au-Dend isotherms suggest thermally induced reorganization of the monolayer, consistent with the morphological changes visualized by TEM. The lift-off point in a π–*A* isotherm represents the onset of lateral interactions as NPs begin to pack at the interface. The observed temperature-dependent increase in the area per particle at the lift-off point and decrease in surface pressure at the transition point for Au-Dend can be attributed to the evolution of the monolayer structure from isotropic islands to 2D networks. These network structures inherently exhibit greater void space and thus require a larger total interfacial area compared to isolated islands at the same particle number.

To quantify this effect, we calculated the porosity (ϕ) of each monolayer using the following expression:

$$\phi =1-\frac{A_{\mathrm{calc}}}{A_{\exp}}$$

where *A*_calc_ is the theoretical close-packed area based on a hexagonal lattice, and *A*_exp_ is the experimental area per particle at a surface pressure of 10 mN m^–1^. The values of *A*_calc_ were estimated as 153.0 nm^2^ for Au-Dend and 55.4 nm^2^ for Au-DT, based on their respective center-to-center distances obtained from TEM. The resulting porosity values are plotted in Figure 3d (red symbols). Notably, the initial negative value for Au-DT may be attributed to the formation of bilayers, as suggested by a transition feature in the isotherm around that pressure. Excluding this outlier, the porosity of the Au-DT monolayer exhibited consistently low porosity (∼20%), whereas Au-Dend remained high (∼80%) across all temperatures, consistent with the presence of lateral voids observed in TEM images. As evident from the TEM observations, the Au-Dend monolayer already exhibits sparsely distributed island-like domains at rt, resulting in a relatively high overall porosity of the film. Consequently, the change in porosity upon heating remains modest, amounting to approximately 5%. In contrast, the *A*_exp_ values at π = 10 mN m^–1^ vary significantly with temperature, supporting a pronounced lateral structural transformation from island-like domains to network-like arrangements. For porosity values calculated using alternative definitions of *A*_exp_ (other than at 10 mN m^–1^), see Figure S8 and Table S1.

The lateral mechanical rigidity of the monolayers was further evaluated using the compressibility modulus $C_{s}^{-1}$ , defined as

$$C_{s}^{-1}=-A\left(\frac{d\pi }{dA}\right)$$

A decrease in $C_{s}^{-1}$ indicates a transition to a more fluid-like and expanded monolayer, while an increase reflects condensation into a less compressible, solid-like phase. $C_{s}^{-1}$ was calculated from the slope of each isotherm between the lift-off and transition points and is plotted in Figure 3d (blue symbols). Although the absolute values of $C_{s}^{-1}$ differ between Au-Dend and Au-DT due to differences in ligand thickness and area per particle, the temperature dependence provides meaningful comparative insight. For Au-Dend, $C_{s}^{-1}$ decreased with increasing temperature, indicating enhanced flexibility and structural fluidity. This behavior agrees with the formation of thermally responsive network structures and further indicates a thermotropic LC transition of dendrons. In contrast, Au-DT exhibited minimal temperature dependence, consistent with a rigid and densely packed monolayer. In addition, the larger slopes (dπ/d*A*) observed in the isotherms of Au-DT compared to those of Au-Dend further support the conclusion that Au-DT form a more rigid monolayer than Au-Dend. These findings collectively demonstrate the thermoresponsive rearrangement capability of the LC Au-Dend monolayer, enabling structural tuning of 2D assemblies of NPs via temperature and lateral compression.

### 2.3 Out-of-Plane Structural Characterization by XRR

XRR is sensitive to the electron density profile perpendicular to the interfaces and thereby probes the vertical distribution of NPs. To investigate structural reorganization during compression, XRR measurements were performed on Au-Dend and Au-DT monolayers under continuous compression in a vibration-free environment. For presentation, the experimental reflectivity (*R*) was normalized to the Fresnel reflectivity (*R*_F_) of an ideally flat air/water interface.

The XRR profiles of the Au-Dend monolayer display broad and weak Kiessig fringes (Figure 4a), which are attributed to the thick and diffuse dendron ligand shell that reduces the electron density contrast at the interface. Similar broad fringes have been reported in systems with small NP cores relative to thick organic shells or in monolayers of NPs grafted with soft polymer/dendrimer ligands.^28^

**Figure 4:**
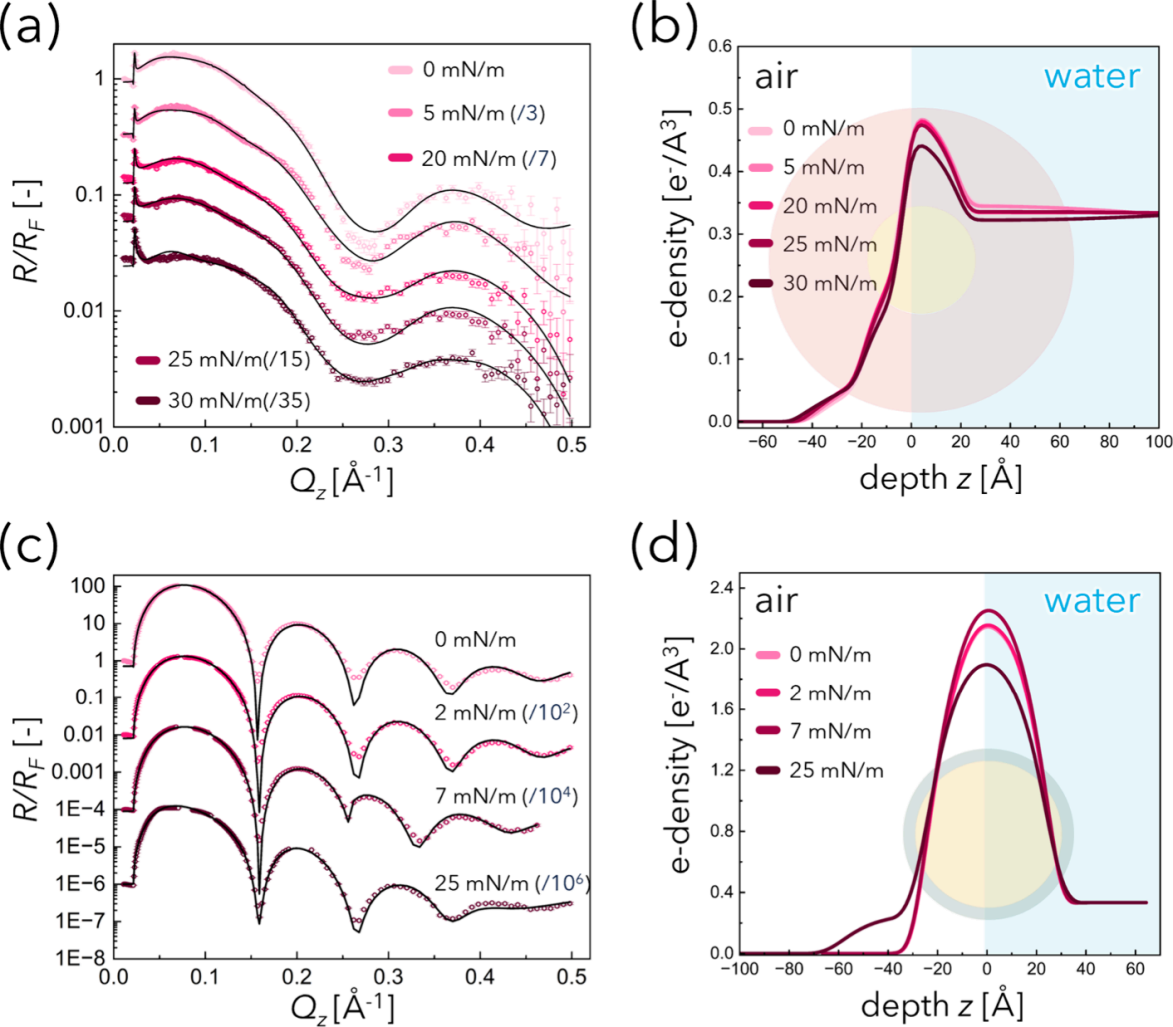
X-ray reflectivity (XRR) profiles, *R*/*R*_F_, for (a) Au-Dend and (c) Au-DT monolayers at various lateral pressures at room temperature. For clarity, the curves were vertically shifted by dividing the intensities by factors of 3 (5 mN m^–1^), 7 (20 mN m^–1^), 15 (25 mN m^–1^), 35 (30 mN m^–1^) in (a), and by factors of 10^2^ (2 mN m^–1^), 10^4^ (7 mN m^–1^), 10^6^ (25 mN m^–1^) in (b). Reconstructed electron density profiles for (b) Au-Dend and (d) Au-DT obtained from the best fits to the data shown in (a) and (c), respectively (black solid lines). The schematic illustrations in (b) and (d) depict representative Au NP cores (yellow spheres) and organic shells (red or green), aligned with the corresponding depth axis (*z*-axis) scale.

The XRR data were analyzed using a generalized spherical core–shell model that allows for nonconcentric core–shell geometries, partial surface coverage, and a vertical offset, Δ_Au/wat_, of the core center with respect to water surface. The details of the model are presented in the Experimental Section in the Supporting Information. To model the reflectivity curves, the continuous electron density profiles that follow from this model were discretized into 1 Å thin slabs and treated with the Parratt formalism.^31^ The optimized model parameters for each pressure are summarized in Table S2. For consistency, the surface coverage *f* was assumed to increase monotonically upon compression.

For Au-Dend, the core radius *r*_Au_ was fixed at the value obtained in the fit at 0 mN m^–1^. Notably, the effective core radius of Au-Dend estimated from XRR fitting was 2.3 nm (yellow circle in Figure 4b), which means 4.6 nm in diameter, smaller than the TEM-measured diameter. This underestimation likely results from the moderate electron density gradient between the dendron ligands and the Au core, and from the significant roughness of the air/water interface (characterized by a height standard deviation parameter σ = 0.3 nm).

Because the fitted electron density of the organic shell (ρ_org_) is slightly higher than that of the water subphase, the depth profiles are sensitive to ligand redistribution across the interface. The electron density profiles in Figure 4b show that, while the monolayer structure is maintained in Au-Dend at all pressures, the electron density distribution originating from the dendrons progressively shifts from the water-facing side toward the air-facing side upon compression. Consistently, the parametrized fits yield a monotonic decrease in ρ_org_ together with a gradual increase in the apparent ligand-shell thickness, *d*_org_ (from 3.9 nm at 0 mN m^–1^ to 5.9 nm at 30 mN m^–1^). These trends indicate a broadening and upward displacement of the organic electron density distributions reflecting an out-of-plane conformational change of the dendrons.

We interpret this evolution as a compression-induced “unfold-stand-up” process: lateral interdigitation is progressively released, dendrons extend and tilt/stand up, and a fraction of the organic layer is displaced toward the air side. This interpretation is supported by our previous SAXS result that the dendron forms spherical assemblies where their amino terminals are the center, with a diameter of ∼9.8 nm in bulk (fully extended length ≈4.9 nm), implying that higher surface pressure drives the ligands toward more extended conformations. It is well established that lateral compression at liquid interfaces promotes to “standing up” transitions of hydrophobic chains repelled out of the water often accompanied by phase segregation of two kinds of ligands and enrichment toward the air side.^32–34^ In the fits we also introduced a vertical offset between the centers of the core and of the shell (Δ_Au/org_) to allow for core–shell asymmetry. The increase of Δ_Au/org_ (from 2.0 nm at 0 mN m^–1^ to 3.4 nm at 30 mN m^–1^) with compression indicates that the Au core becomes displaced toward the water-facing side relative to the organic ligand shell, suggesting the development of an anisotropic core–shell geometry. Since XRR primarily probes the out-of-plane structure, complementary GISAXS measurements are required to resolve the associated in-plane reorganization.

In contrast, the XRR profiles of the Au-DT monolayer exhibited sharp and well-defined fringes (Figure 4c), indicating a dense and rigid monolayer.^35,36^ For Au-DT, *r*_Au_, *d*_org_, and ρ_org_ were fixed based on fitting at 0 mN m^–1^, and Δ_Au/org_ was set to zero throughout, as the rigid DT shell was assumed to prevent significant core movement. The particle diameter extracted from the XRR profile was 6.0 nm (yellow circle in Figure 4d), closely matching the TEM diameter, further confirming the compact and well-defined architecture of the Au-DT monolayer.

Compression of the Au-DT monolayer led to an increase in core-associated electron density, reflecting increased surface coverage. At 25 mN m^–1^, the electron density profile suggested the onset of bilayer formation, as some NPs appeared to stack atop the monolayer.

In summary, XRR measurements revealed a clear contrast between Au-Dend and Au-DT in response to compression. The Au-Dend monolayer displayed flexibility and probability of dynamic conformational change of organic shell to anisotropic structure, whereas Au-DT formed a rigid, well-packed layer with little conformational change.

### 2.4 In-Plane Structural Characterization by GISAXS

Synchrotron GISAXS measurements were conducted to elucidate the in-plane organization of the NP monolayers. Figure 5 shows the 2D GISAXS patterns and corresponding linecut profiles along *Q_z_* = 0.1 Å^–1^ at various surface pressures for Au-Dend at 21.2 and 40.7 °C, and for Au-DT at 21.2 °C. The black line in Figure 5b represents a simulated profile based on a 2D hexagonal lattice at the air/water interface, assuming hemispherical Au NPs partially submerged in the subphase. The simulation was performed using BornAgain,^37^ with parameters set as NP diameter = 6.5 nm, center-to-center distance = 11.0 nm, decay length = 25.0 nm, and position variance = 1.5 nm to reproduce the pattern for Au-Dend at rt, 0 mN m^–1^ (see Table 1). All experimental peak positions matched well with the simulated 2D hexagonal lattice, confirming that both Au-Dend and Au-DT qualitatively maintained such in-plane ordering throughout compression, albeit with some variations. In this *Q_xy_* range, the GISAXS patterns capture the short-range ordering of NPs over several-nanometer length scales, reflecting crystallinity and structural coherence in the local hexagonal packing rather than the macroscopic domain morphologies (e.g., island-like or network-like) observed in TEM.

**Figure 5:**
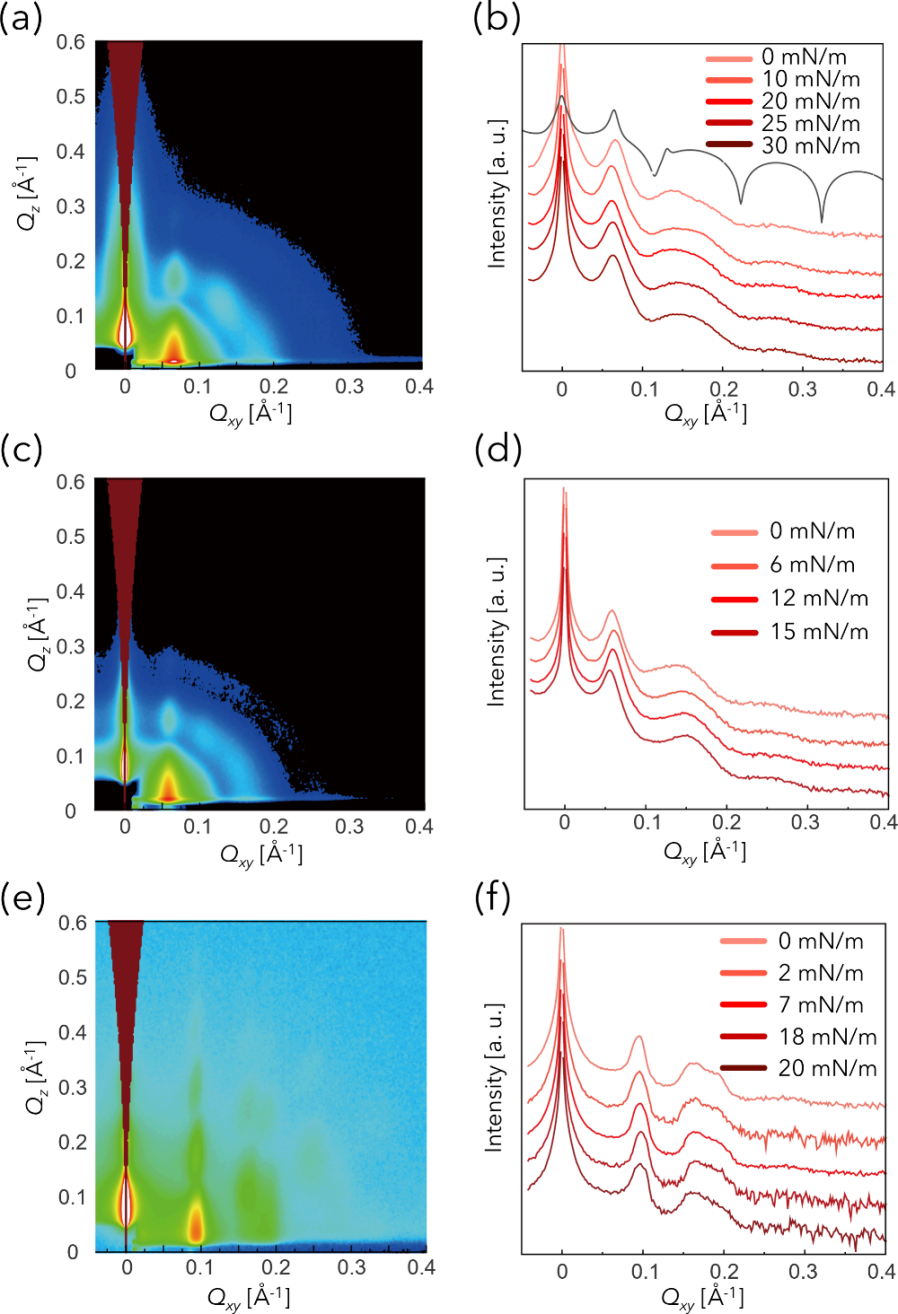
GISAXS patterns of Au-Dend monolayers at water temperature of (a) room temperature, (c) 40.7 °C, and (e) Au-DT monolayers at room temperature, shown as functions of *Q*_*xy*_ and *Q*_*z*_. All patterns were acquired at the air/water interface at a surface pressure of 0 mN m^–1^. Intensities are displayed on a logarithmic scale. (b), (d), and (f) show 1D linecut profiles extracted from the GISAXS patterns along *Q*_*z*_ = 0.1 Å^–1^ at various lateral pressures. The black line in (b) corresponds to a simulated scattering profile based on a 2D hexagonal lattice with a center-to-center distance of 11.0 nm. GISAXS patterns at other surface pressures are provided in Figure S9.

**Table 1:** Summary of Structural Information of Au-Dend and Au-DT Monolayers Extracted from GISAXS Patterns at Various Surface Pressures

sample	temperature (°C)	surface pressure (mN m^–1^)	*Q*_10_ (Å^–1^)	*a* (nm)	the center-to-center distance in TEM image (nm)	fwhm_*Q*(10)_ (Å^–1^)	domain size (nm)	number of NPs
Au-Dend	21.2	0	0.0660	11.0	11.0 ± 1.5	0.0232	27.0	2.5
		10	0.0615	11.8		0.0233	26.9	2.3
		20	0.0615	11.8	10.6 ± 0.7	0.0257	24.5	2.1
		25	0.0630	11.5		0.0250	25.1	2.2
		30	0.0630	11.5		0.0257	24.5	2.1
		60			11.4 ± 1.0			
	40.7	0	0.0585	12.4	12.2 ± 1.1	0.0217	29.0	2.3
		6	0.0600	12.1		0.0226	27.8	2.3
		10			12.2 ± 1.1			
		12	0.0585	12.4		0.0208	30.3	2.4
		15	0.0555	13.1		0.0244	25.8	2.0
		30			12.2 ± 1.5			
Au-DT	21.2	0	0.0960	7.6	7.4 ± 0.3	0.0139	45.2	6.0
		2	0.0960	7.6	7.7 ± 0.1	0.0150	42.0	5.6
		7	0.0975	7.4	7.5 ± 0.3	0.0159	39.6	5.3
		18	0.0975	7.4		0.0148	42.5	5.7
		20	0.0960	7.6		0.0177	35.4	4.7

The lattice constant a of each 2D hexagonal structure was determined from the *Q*_(10)_ peak position and is summarized in Table 1. Because a directly corresponds to the in-plane center-to-center distance of NPs in a 2D hexagonal assembly, the values agree well with the center-to-center distances obtained from TEM. Notably, the standard deviations of a were smaller for Au-DT than Au-Dend, indicating its higher structural uniformity.

Additionally, the sharper and more intense scattering peaks observed for Au-DT reflect their higher degree of in-plane crystallinity. Domain sizes—defined as the coherence length over which the 2D hexagonal arrangement persists—were estimated from the full width at half-maximum (fwhm) of the *Q*_(10)_ peaks using the relation *L* = 2π/fwhm_*Q*(10)_.^20,21^ The domain sizes of the Au-Dend monolayer were approximately 30 nm, corresponding to only 2–3 NPs, indicating the presence of small domains. In contrast, the domain sizes of the Au-DT monolayer were about 45 nm, reflecting well-defined ordering extending over ∼6 NPs. These values are consistent with the ordered regions visually identified in TEM images.

In self-assembled NP films, particularly those composed of NPs with small cores and bulky ligand shells, van der Waals interactions are weaker and often lead to short-range order spanning only 2–3 NPs.^38^ Similarly, such small domain has been reported in a suspension system.^39^ On the other hand, monolayers formed from rigid ligands with the diameters around 6 nm have been reported to form crystalline domains extending over 5–6 NPs or more as stronger van der Waals attraction is exerted in such rigid monolayers, consistent with the results in this work.^40,41^

This result supports the conclusion that Au-DT forms stable, solid-like monolayers, while Au-Dend exhibits more flexible, liquid-like behavior. The agreement between the local, short-range order observed in TEM and the average long-range ordering inferred from GISAXS demonstrates that these two techniques complementarily reveal the in-plane structure of the NP monolayers at the air/water interface.

For Au-Dend, the systematically larger *a* observed at 40.7 °C compared to rt suggest that the dendrons adopt a more extended conformation in the lateral direction, thereby imparting anisotropy to the overall particle geometry. Moreover, even though the lateral pressure increases, the lattice constant *a* does not show any measurable decrease at either rt or 40.7 °C. As discussed in the Section 2.3, we propose that the dendron ligands initially lie flat on the interface during the gas-like phase, then progressively stand up and extend their alkyl chains vertically toward air-facing side upon compression—analogous to conformational transitions typically observed in Langmuir monolayers of hydrophobic molecules.^42,43^ Further discussion of this coupled in-plane and out-of-plane conformational change is provided in the following section.

### 2.5 Temperature- and Pressure-Driven Transition Mechanism of NP Assemblies

Finally, we discuss the structural reorganization of the Au-Dend monolayer upon heating and compression, integrating the structural insights obtained from X-ray measurements.

Figure 6 presents a phase diagram summarizing the organizational states of Au-Dend at the air/water interface as functions of temperature and surface pressure. TEM observations revealed that, unlike Au-Dend, Au-DT exhibited negligible temperature-dependent structural changes and instead underwent island coalescence upon compression, corresponding to the typical formation process of a solid Langmuir monolayer. On the other hand, Au-Dend underwent a sequence of transitions from isolated NP islands to 1D chains and, ultimately, to 2D network structures upon heating. Furthermore, compression led to the reappearance of island domains even at higher temperatures. We attribute these temperature- and pressure-induced transformations to the dynamic conformational redistribution and conformational change of organic ligands on the NP surface, which leads to corresponding changes in the lateral isotropy of the NP shape.

**Figure 6:**
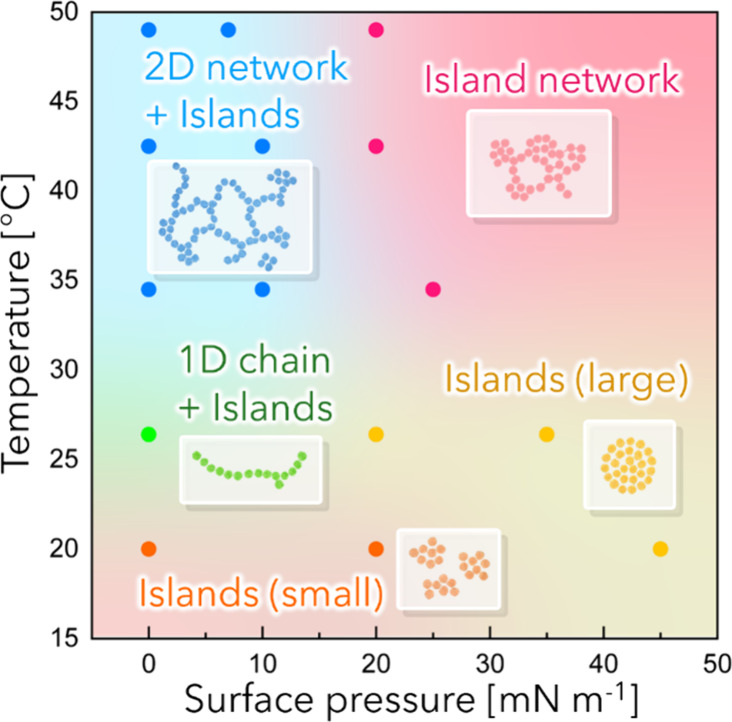
Phase diagram of Au-Dend monolayers at the air/water interface, plotted as a function of surface pressure and subphase temperature, reconstructed from the TEM images in Figure 2c. Distinct structural regimes are identified: small islands, 1D chain-like assemblies with islands, extended 2D networks with islands, larger compact islands, and island-like networks. The schematic illustrations highlight the representative self-assembled morphologies observed in each region. This diagram summarizes the temperature- and pressure-dependent structural transitions of the interfacial NP assemblies.

Before spreading, Au-Dend dispersed in an organic solvent is solvated, and both the dendron and dodecanethiol ligands on the NP surface are expected to extend radially from the Au core (Figure 7a). Interestingly, XRR analysis revealed that Δ_Au/org_ was 2.0 nm at 0 mN m^–1^, indicating that the Au core was displaced toward the water-facing side relative to the organic ligand shell. This configuration forms a vertically anisotropic but laterally isotropic particle shape, as illustrated in Figure 7b­(i). Such a laterally isotropic shape promotes the formation of isotropic two-dimensional assemblies (Figure 7b­(ii)), namely, island-like structures as macroscopic arrangements.

**Figure 7:**
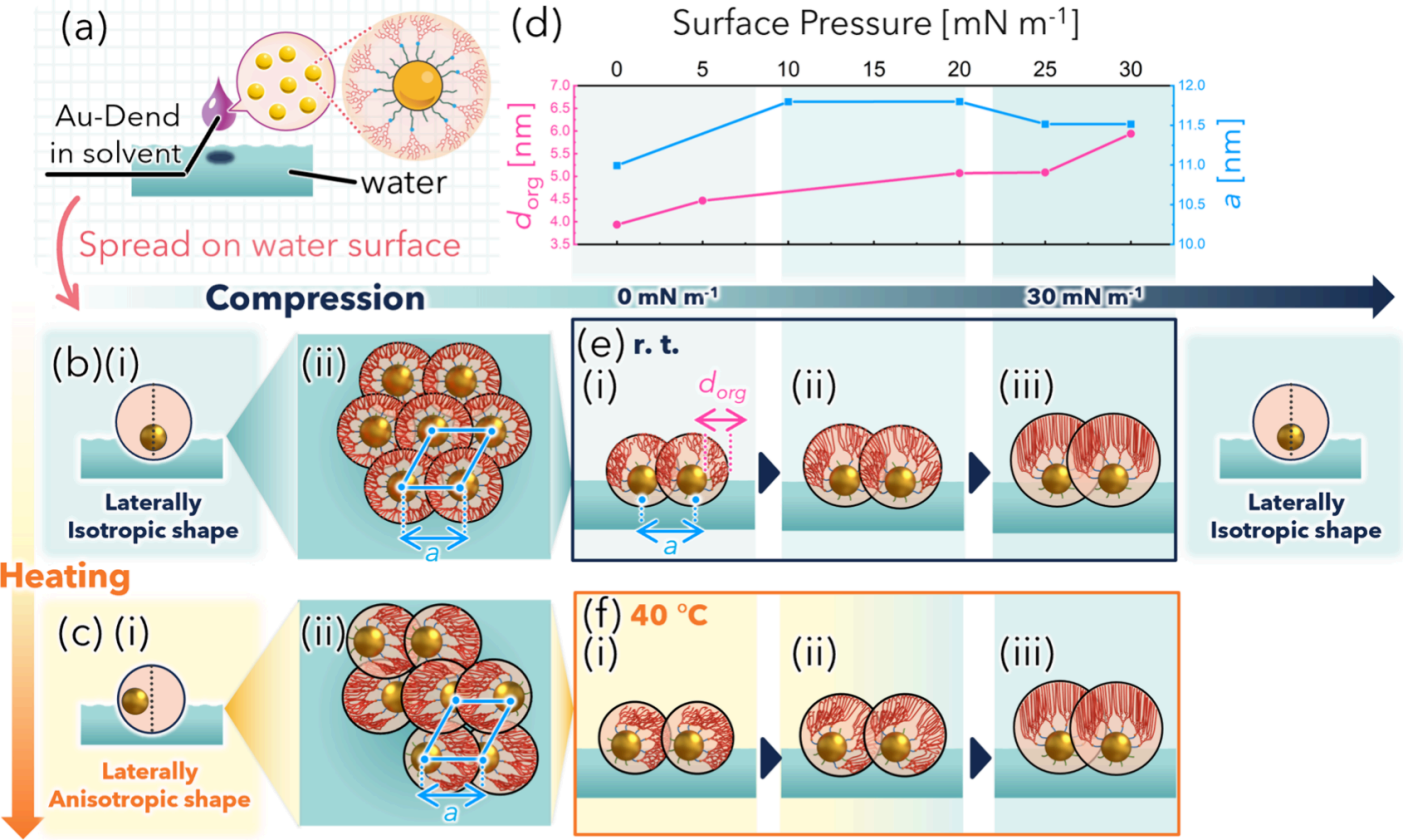
Schematic illustration of the temperature and pressure-induced structural reorganization of Au-Dend monolayer at the air/water interface. (a) Schematic of Au-Dend when spread on the water surface. The dendron and dodecanethiol ligands are expected to be solvated by the organic solvent and extend radially from the Au core. (i) Side and (ii) top views illustrating particle configurations at (b) rt and (c) 40 °C. The pink circles represent the organic shell. According to XRR analysis, at rt, the Au core is slightly displaced toward the water side within the organic shell, resulting in a vertically anisotropic but a laterally isotropic particle shape. The laterally isotropic particle shape leads to isotropic hexagonal island-like domains. At elevated temperatures, π–π stacking among the aromatic groups of dendrons induces a lying-down conformation, resulting in a laterally anisotropic particle shape. Anisotropic particle shapes favor directionally anisotropic network structures. (d) Evolution of the organic ligand thickness (*d*_org_) obtained from XRR analysis and the lattice constant (*a*) from GISAXS at rt as functions of surface pressure. *d*_org_ increased with compression, while becoming nearly constant at higher surface pressures. Side-view schematics of ligand conformational changes during compression at (e) rt, (i) 0 mN m^–1^, (ii) 20 mN m^–1^, (iii) 30 mN m^–1^, and (f) 40 °C, (i) 0 mN m^–1^, (ii) 10 mN m^–1^, (iii) 30 mN m^–1^. At rt, interdigitated dendron chains progressively extend while maintaining lateral isotropy, whereas at 40 °C, the laterally anisotropic particles gradually transform into isotropic ones with compression—corresponding to the transition from network-like to island-like assemblies. The pink and blue arrows indicate *d*_org_ and *a*, respectively. The light-blue backgrounds in (b), (e), and (f) denote laterally isotropic particle shapes, where the darker shades in (e) represent the progression of compression corresponding to the three compression stages shown by the shaded regions in (d). The yellow backgrounds in (c) and (f) indicate laterally anisotropic particle shapes.

#### 2.5.1 Temperature-Induced Ligand Redistribution

In contrast, upon heating, the Au-Dend monolayer transitioned into anisotropic network assemblies rather than island-like ones. In our previous study on dendronized CdS quantum dots,^17^ redistribution of dendron molecules and dodecanethiol ligands on the NP surface led to a transformation of the overall particle shape from an isotropic to an anisotropic configuration as the temperature increase (Figure 7c­(i)). This reconfiguration is likely stabilized by π–π stacking among dendrons, enabling the formation of a low-symmetry *P*2_1_3 superlattice.

At the air/water interface, a similar mechanism is likely operative: the lying-down conformation of dendrons, arising from temperature-induced lateral redistribution of dodecanethiols and dendrons, produces a laterally anisotropic particle shape, which in turn favors directionally dependent chain-like and network assemblies (Figure 7c­(ii)). Importantly, the laterally extended dendrons can still interdigitate with those on neighboring particles, allowing the monolayer to retain local hexagonal registry confirmed by GISAXS, even as its macroscopic arrangement shifts toward anisotropic network structures. This interpretation is further supported by the larger lattice constant *a* at 40 °C compared to rt, indicating laterally extended ligand conformations consistent with anisotropic interparticle spacing.

Such ligand-induced anisotropy is known to generate directional interactions and has been reported in other NP systems featuring anisotropic ligand distributions or particle shapes.^19,44–46^ The present results well agreed with these reports as the dendron shell of Au-Dend became anisotropic upon heating, the assemblies evolved into network-like structures. Additional TEM images at 27.9 °C and 0 mN m^–1^ (Figure S10) suggest the coexistence of 1D chains and discrete island domains, consistent with an intermediate state between isotropic (rt) and fully anisotropic (≥40 °C) assemblies.

Notably, while the conventional solid films of dendronized Au NPs deposited on substrates exhibited a phase transition above 100 °C, accompanied by changes in their 3D assemblies, the present study demonstrates that analogous transitions can occur at much lower temperatures at the air/water interface, where the organic ligands possess greater conformational flexibility. Achieving phase transitions at near-physiological temperatures highlights the unique advantage of interfacial architectures and points to promising opportunities for biomimetic and biomedical applications.

#### 2.5.2 Pressure-Induced Conformational Change

TEM observations of the Au-Dend monolayer revealed that compression induced coalescence of isotropic islands at rt, similar to the behavior observed for Au-DT. Figure 7d illustrates the evolution of *d*_org_ obtained from XRR analysis and the in-plane lattice constant *a* determined by GISAXS, which corresponds to the proposed interfacial reconfiguration of dendrons at rt (Figure 7e). As illustrated in Figure 7e­(i), in the absence of compression (0 mN m^–1^), Au-Dend resides at the air/water interface with its bulky hydrophobic dendron ligands partially oriented toward the air side. At moderate compression (Figure 7e­(ii)), the dendron molecules progressively tilt upward toward the air side while repelling each other laterally. This conformational change accounts for the observed increase in *d*_org_ and the fact that *a* does not decrease upon lateral compression (see Figure 7d). At higher compression (Figure 7e­(iii)), the alkyl chains of dendrons adopt a more brush-like conformation and become vertically oriented toward the air side, characteristic of a liquid-condensed state. Throughout this process at rt, the assemblies retain lateral isotropy, consistent with the continuous formation of island-like domains typical of Langmuir films.

In contrast, at elevated temperatures, compression transformed anisotropic network structures back into more isotropic island-like arrangements. As described in the previous section, Au-Dend adopts an anisotropic shape above 40 °C due to the spontaneous rearrangement of dendrons and dodecanethiols. In this state, dendrons that were isotropically distributed near the interface at rt became redistributed to one side, laterally oval-like configuration by π–π stacking stabilization (Figure 7f­(i)). This anisotropic particle shape explained the formation of network-like assemblies at higher temperatures. With further compression (Figure 7f­(ii,iii)), the dendrons gradually stand more upright and the particle shapes approach lateral isotropy, resulting in the re-emergence of island-like arrangements. Thus, compression counteracts the thermally induced anisotropy, ultimately driving the system back toward isotropic packing.

Such temperature- and compression-driven reorganizations of ligand conformations have also been reported for PNIPAM-functionalized NP systems.^24,26^ In these cases, PNIPAM chains on the NP surface were shown to change from a so-called “pancake”-like conformation to a brush-like structure upon compression, and from an extended to a contracted state upon heating to ∼30 °C. However, these conformational changes in PNIPAM ligands did not result in a dynamic reorganization of the NP assemblies, highlighting that the intrinsic self-organizing ability of dendrons plays a key role in enabling multiple structural arrangements of NPs. Additionally, the precise surface design of NPs using two distinct ligands, LC dendrons and rigid dodecanethiols, enabled the emergence of anisotropic particle shapes and the direct observation of stimuli-responsive behavior of ligands at the NP interface. In our study, heating increases particle-shape anisotropy, driving a transition from island-like to network-like assemblies, whereas compression restores isotropy, favoring island formation. Furthermore, the increase in area per particle at the lift-off points observed in the isotherms of Au-Dend at elevated temperatures is in good agreement with the formation of laterally isotropic conformations of dendrons on the Au-Dend surface. This mechanistic picture is fully consistent with the TEM, XRR, and GISAXS observations.

Such dynamic and flexible behavior is absent in Au-DT, whose rigid linear alkyl ligands remain isotropic and relatively static under the same conditions. In Au-DT monolayers, the absence of directional interparticle interactions results in isotropic packing, with compression simply densifying the film via coalescence of islands.

In summary, we elucidated the temperature- and surface pressure-dependent reorganization of dendronized Au NPs at the air/water interface, driven by conformational adaptability of their ligand shells. This dynamic ligand redistribution is enabled by a precisely designed NP surface, dual-functionalized with liquid-crystalline dendrons and simple alkanethiols. The observed ligand redistribution-driven anisotropy is not a generic feature of dendritic ligands but arises from the precise surface engineering employed in this study. Our previous work demonstrated that only second-generation dendrons, combined with an appropriate fraction of DT, enables thermally induced structural transitions in bulk NP assemblies, whereas first- and third-generation dendrons did not produce well-defined ordered phases. Together with the influence of ligand volume, chain length, and NP core curvature on surface redistribution, these findings suggest that an optimal dendron generation and composition are required to induce the dynamic interfacial reorganization reported here.

Whereas most previous studies on interfacial NP assemblies have focused on static arrangement formation, our results show for the first time that stimuli-induced redistribution of two coexisting ligands can actively drive rearrangements of the NP assemblies themselves at the interface. Furthermore, by integrating TEM, isotherms, and both in-plane and out-of-plane X-ray analyses, we provide a comprehensive description that connects local structural changes with long-range ordering. We anticipate that this combined approach will serve as a benchmark for the investigation of other stimulus-responsive interfacial materials in the future.

## 3 Conclusion

In this study, we compared Au-Dend, functionalized with flexible and thermoresponsive dendron ligands, with Au-DT, functionalized with rigid linear alkyl chains, to elucidate the ligand-dependent self-organization of NP monolayers. Au-DT exhibited the typical behavior of NP-based Langmuir films, where isotropic, island-like domains coalesce upon compression. In contrast, Au-Dend formed a monolayer similar to Au-DT at rt, but TEM observations revealed a gradual transition from island-like arrays to anisotropic network structures upon heating to ∼50 °C. Compression after heating induced the re-emergence of island-like domains within the network, suggesting that the interfacial conformation of Au-Dend at rt was reproduced by lateral compression.

Temperature-dependent π–*A* isotherms reflected these morphological changes: at rt, Au-Dend displayed a steep rise in surface pressure, similar to Au-DT, while both the lift-off point and the transition point systematically shifted with heating. These features are consistent with an increase in monolayer flexibility upon thermal transformation into a network-like arrangement. Comprehensive X-ray analysis further revealed that both the isotropy and anisotropy of the NPs, including the ligand shells, varied during compression. At elevated temperatures, dendron and dodecanethiol ligands rearranged on the NP surface, imparting anisotropy to the particle shape and promoting the formation of anisotropic arrangements. Upon further compression, the dendrons reoriented vertically toward the water surface, restoring lateral isotropy and reforming island-like structures.

In our previous studies, the same dendron/alkanethiol surface design endowed Au NPs,^16^ CdS quantum dots,^17^ and FePt NPs^18^ with LC character, enabling thermally induced phase transitions and lattice control in the dry state. In particular, dendron-functionalized Au NPs represented the first example of spherical NPs self-assembling into a simple cubic lattice. In contrast to these bulk systems, the present study demonstrates that when confined at the air/water interface, the same surface design gives rise to a distinct ordering principle, namely dynamic interfacial reorganization driven by ligand-mediated particle-shape anisotropy. Unlike the discrete phase transitions observed in the dry state, the interfacial system exhibits continuous and flexible structural evolution, highlighting how deployment of the same surface-engineered NPs in a different physical environment leads to emergent assembly behavior.

Overall, we have developed a liquid-like NP film capable of reorganizing its 2D arrangement in response to temperature and lateral pressure at the air/water interface. This work thus highlights a key distinction: while PNIPAM ligands undergo conformational transitions without reorganizing NP assemblies, precise dual-functionalization with thermoresponsive, flexible dendron and rigid alkanethiol endow NPs with the unique ability to transform their 2D architectures in response to external stimuli. The interfacial phase transition elucidated here, driven by ligand-derived anisotropy, provides key design principles for interface-active NP materials via ligand engineering. Furthermore, NP assemblies capable of thermally switching between dense and void-rich arrangements near physiological temperatures hold potential for biomedical applications, such as drug delivery systems targeting cancer cells, which are often slightly warmer than surrounding tissues. Looking forward, functionalization with ligands responsive to additional stimuli—such as ionic strength or pH—could enable a versatile platform for adaptive 2D NP materials. Unlike the rigid films that have been predominantly reported, liquid-like membranes composed of inorganic NPs yet capable of structural rearrangements can function as adaptive smart surfaces that optimize their on/off states responses to environmental stimuli, offering a new conceptual framework for materials in microfluidics. The thermoresponsive NP monolayer system demonstrated here represents a foundational step toward such multifunctional interfacial nanomaterials.

## Supplementary Material



## Notes

Additional experimental details, theoretical calculation method, structural formula of the dendron, TEM images of as-synthesized Au-Dend and Au-DT, enlarged TEM images and fast Fourier transform patterns of Figure 2c and d, summary of the fitting parameters of XRR, GISAXS patterns of Au-Dend and Au-DT monolayer at various surface pressures, TEM images of Au-Dend monolayers at 27.9 °C and 0 mN m^–1^, characterization of Au-Dend, and additional references (PDF)
