# Supplementary material for: Temperature- and Pressure-Induced Ligand Anisotropy Drives Structural Reorganization of Dendronized Gold Nanoparticle Monolayers
Source: J Am Chem Soc. 2026 May 1;148(18):18769–81. doi: 10.1021/jacs.5c22437 (PMC13185118; doi:10.1021/jacs.5c22437)
Supplement: Supplementary file 1 [file ja5c22437_si_001.pdf]

Supporting Information

Temperature- and Pressure- Induced Ligand Anisotropy Drives Structural Reorganization of Dendronized Gold Nanoparticle Monolayers

Rina Sato[*1,2], Joshua Reed[2], Emanuel Schneck[*2], Kiyoshi Kanie[*1,3]

1: Institute of Multidisciplinary Research for Advanced Materials, Tohoku University,
2-1-1 Katahira, Aoba-ku, Sendai, Miyagi, 980-8577, Japan.
2: Institute for Condensed Matter Physics, Technische Universität Darmstadt,
Hochschulstrasse 8, 64289 Darmstadt, Germany.
3: International Center for Synchrotron Radiation Innovation Smart, Tohoku University,
2-1-1 Katahira, Aoba-ku, Sendai, Miyagi, 980-8577, Japan.

Email: rina.sato.s5@dc.tohoku.ac.jp; emanuel.schneck@pkm.tu-darmstadt.de; kanie@tohoku.ac.jp

1. Experimental Section

Materials.

Hydrogen tetrachloroaurate(III) tetrahydrate was purchased by Wako Pure Chemical Industries. 1-dodecanethiol, borane-tert-butylamine complex, and mercaptohexadecanoic acid were supplied by Sigma–Aldrich. Octyl ether was purchased from Tokyo Chemical Industry. All the chemicals were used without further purification. Dendron was synthesized following the previous report[1].

Sample preparation.

Dendronized Au NPs were prepared by following the procedure in ref[1]. The molar ratio of 16-mercaptohexadecanoic acid (MHA) to 1-dodecanethiol (DT) on the NP surface was 3:2. Fourier transform infrared spectroscopy measurement (Figure S11) and thermogravimetric analysis (Figure S12) to determine the modification number of dendron on the NP surface and were performed for further characterization. Au-DT was synthesized following previous methods[2] with some modifications.

The NP monolayers formed on water was observed using transmission electron microscopy (TEM) (HITACHI H-7650, accelerating voltage of 100 kV, emission current of 20 μA) by horizontally transferring them onto TEM grids (STEM, PVF-C15).

Isotherms.

Temperature-dependent π-A isotherms were measured using a Langmuir trough (maximum area: 144 cm^2) with a thermostat (LAUDA, E100). In a typical measurement, hot water coming from the thermostat circulated right under the trough, and 50 μL of Au-Dend (1 mg/mL, chloroform : toluene = 3:1 as a volume fraction) or 80 μL of Au-DT (5 mg/mL, toluene) was dropped 30 min after started circulating to endure the water temperature reached to equilibrium. To ignore the effect of water evaporation as heating above 50 °C, water warmed at 60 °C in 100 mL beakers were put inside a chamber covering the trough. Figure S13 proves that this solution prevented water in the trough from evaporating by increased humidity in the chamber.

X-ray reflectometry (XRR).

XRR measurements were carried out using a D8 Advance reflectometer (Bruker AXS, Karlsruhe, Germany) featuring a vertical goniometer and horizontal sample geometry, allowing the liquid surface to be studied without being disturbed during the measurements. The following descriptions are partially reproduced from ref[3]. A Langmuir trough (KSV 1000, Helsinki, Finland) with one Teflon barrier for asymmetric film compression was enclosed in a box with Kapton windows through which the incident and reflected X-ray beams pass. The dimensions of the trough were 85 mm × 320

mm × 4 mm for a total subphase volume of ≈110 mL when filled to a positive meniscus. Reflectivity curves were measured in the θ–2θ geometry, where θ is the incident angle. A conventional X-ray tube with a Cu anode (Cu Kα, wavelength $\lambda = 1.54$ Å) was used to generate an X-ray beam with a line focus. The beam was monochromized by a Göbel mirror (W/Si multilayer mirror) and collimated through two narrow horizontal slits of 0.1 mm with a switchable absorber (calibrated Cu attenuator) in between. Soller slits ($\Delta\theta_x$ = 25 mrad) were placed after the last horizontal slit and directly in front of the detector. The intensity was recorded with a Våntec-1 line detector (Bruker AXS, Germany). Data were corrected using the known attenuation factors. Finally, the angular reflectivity scans were transformed to reflectivity curves as a function of the perpendicular scattering vector component, $Q_z = 4\pi \sin\theta/\lambda$.

For analysis, the experimental data were compared with theoretically modeled XRR curves based on a spherical core-shell model representation of the electron density profiles of the interfacial NP layers. In this model, NPs were described as core-shell structures with a spherical Au core with radius r_{Au} and an organic ligand shell with thickness d_{org}.

The associated volume fractions of gold and of the organic material are then given as

$$\phi_{\mathrm{Au}}(z) = \frac{\pi(r_{\mathrm{Au}}^2 - z^2)}{A_{\mathrm{np}}}$$

and

$$\phi_{\mathrm{org}}(z) = \pi\frac{\left(r_{\mathrm{Au}} + d_{\mathrm{org}}\right)^2 - \left(z - \Delta_{\mathrm{Au/org}}\right)^2 - (r_{\mathrm{Au}}^2 - z^2)}{A_{\mathrm{np}}}$$

where A_{np} is the area per nanoparticle, as imposed by the coverage fraction *f*, and $\Delta_{\mathrm{Au/org}}$ is the vertical offset between the centers of the core and of the shell. The third ingredient is the volume fraction profile of water, $\phi_{\mathrm{wat}}(z)$, which was described as a sharp step function between 0 and 1-$1 - \left[\phi_{\mathrm{Au}}(z) + \phi_{\mathrm{org}}(z)\right]$, located at the z-position of the water surface, which was allowed to deviate from the center position of the core (at $z = 0$), by an offset $\Delta_{\mathrm{Au/wat}}$. The resulting, idealized electron density profile follows as

$$\rho_{\mathrm{ideal}}(z) = \rho_{\mathrm{Au}}\phi_{\mathrm{Au}}(z) + \rho_{\mathrm{org}}\phi_{\mathrm{org}}(z) + \rho_{\mathrm{wat}}\phi_{\mathrm{wat}}(z)$$

The interfacial roughness of the water surface in terms of capillary waves was taken into account by convolution the idealized electron density profile with a Gaussian function $g(z)$ of width σ:

$$\rho(z) = \rho_{\mathrm{ideal}}(z) \otimes g(z)$$

Where $\otimes$ denotes the convolution operator. The best-matching parameters were then obtained by χ^2-minimization of the deviation between the experimental and theoretical XRR data. A schematic of XRR model parameters for NP monolayers is shown in Figure S12.

Synchrotron-Based Grazing-Incidence X-ray Scattering Techniques.

Grazing-Incidence X-ray Scarrting experiments were conducted at the beamline P08 at storage ring PETRA III of the Deutsches Elektronen-Synchrotron (DESY, Hamburg, Germany). The following descriptions are partially reproduced from ref[4]. The Langmuir trough was located in a hermetically sealed helium-filled container with Kapton windows, and the temperature was kept at 20 °C or 40 °C with the help of a thermostat. The X-ray beam was monochromatized to a photon energy of 15 keV, corresponding to a wavelength of $\lambda = 0.826$ Å. The incident angle was $\alpha_i = 0.07°$, about 85% of the critical angle of total reflection $\alpha_c = 0.082°$ of the interfaces between the two bulk media, air and water. A ground glass plate was placed approximately 0.3 mm beneath the illuminated area of the monolayer in order to suppress vibrations of the water surface.

The scattering signal was collected with a two-dimensional position-sensitive detector (PSD) (Eiger2 1M, sample-detector distance: 560.7 mm) by scanning the azimuth angle 2θ and, with that, the in-plane component $Q_{xy} = (4\pi/\lambda)\ \sin(\theta)$ of the scattering vector $Q = (Q_{xy}, Q_z)^T$. The out-of-plane component, $Q_z = (2\pi/\lambda)[\ \sin(\alpha) + \sin(\alpha_i)]$, is encoded in the vertical position of the PSD channels, where α denotes the angle between the scattered direction and the sample plane. The in-plane beam divergence was reduced with a Soller collimator placed in front of the PSD, providing $\Delta 2\theta \approx 0.09°$ (FWHM), corresponding to $w_{xy}^{res} = (4\pi/\lambda)\ \sin(\Delta 2\theta/2) \approx 0.012$ Å^{-1}.

2. Characterizations

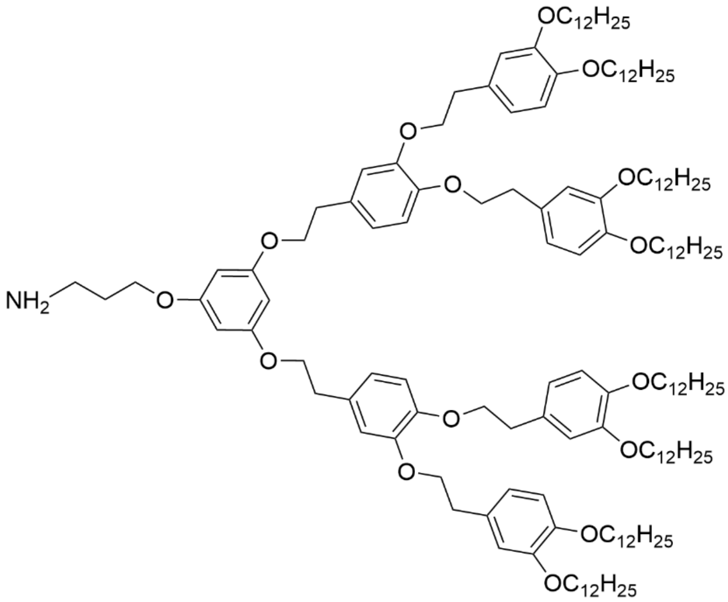

Figure S1 Structural formula of the liquid-crystalline dendron.

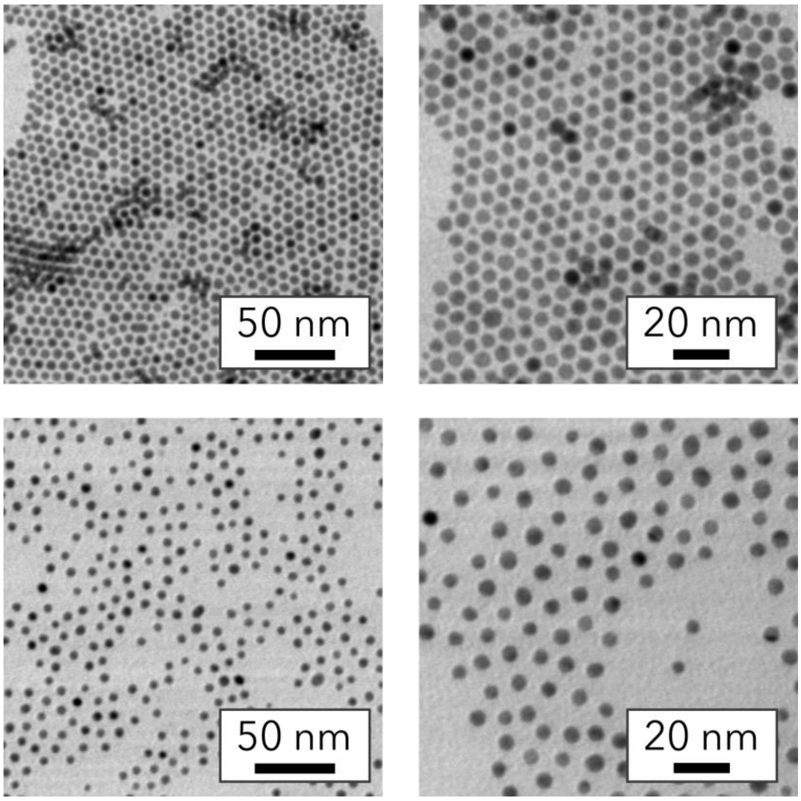

Figure S2 TEM images of as-synthesized Au-DT (top) and Au-Dend (bottom).

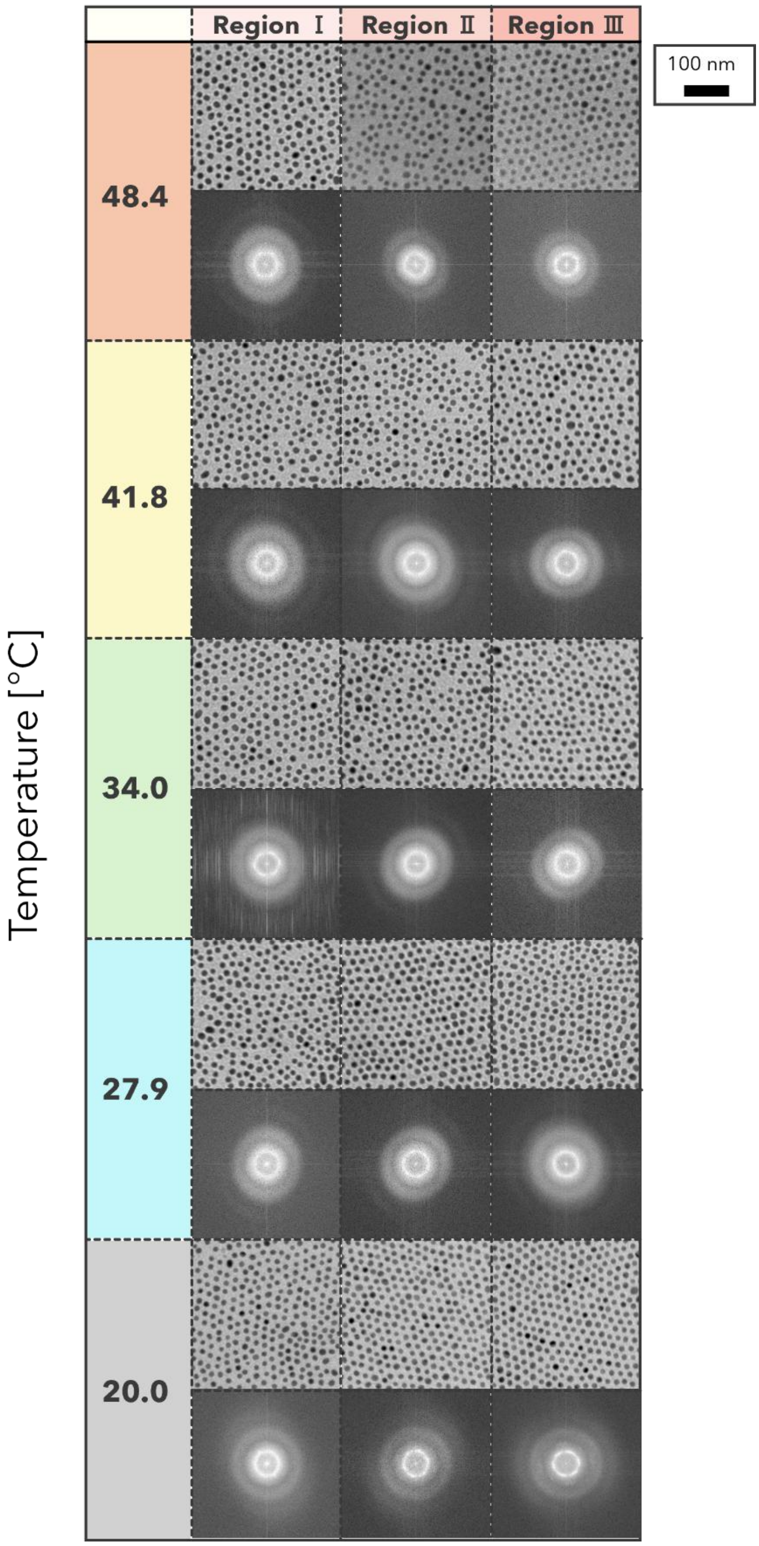

Figure S3 TEM images enlarged from Figure 2c(i) and fast Fourier transform (FFT) patterns of Au-Dend monolayers under various surface pressures and temperatures.

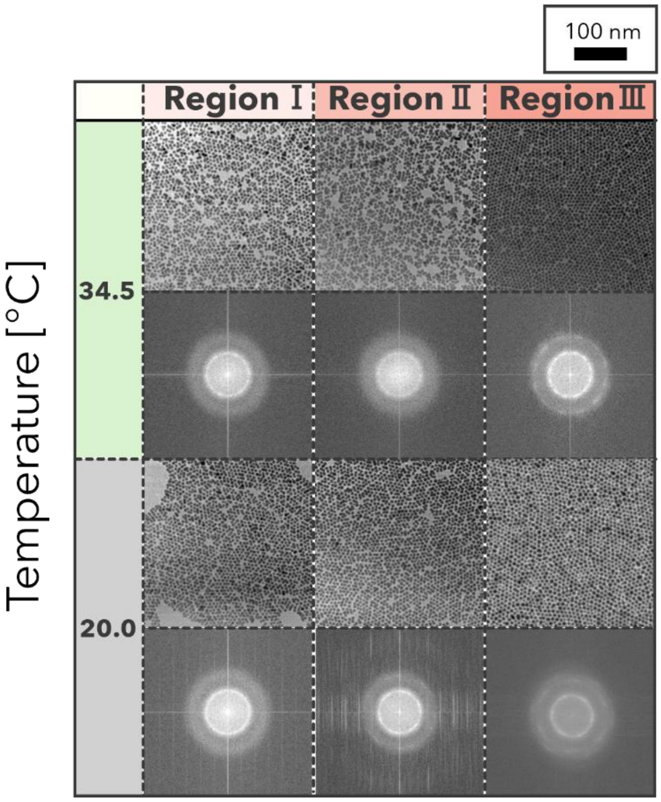

Figure S4 TEM images enlarged from Figure 2c(ii) and FFT patterns of Au-DT monolayers under various surface pressures and temperatures.

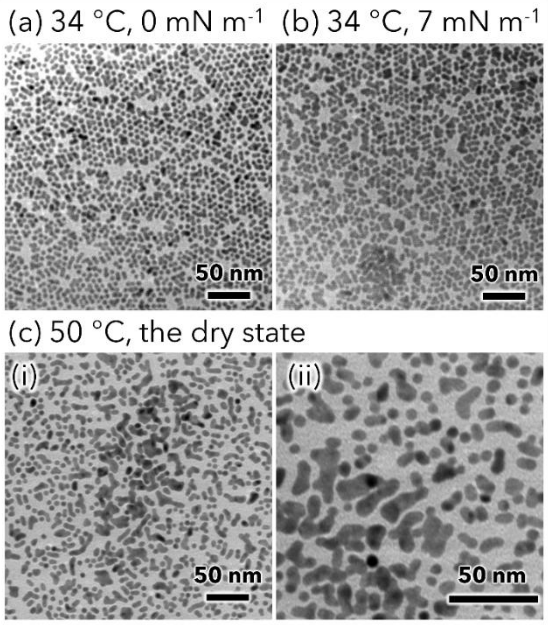

Figure S5 TEM images of Au-Dend monolayers at elevated temperatures: (a,b) at a water temperature of 34 °C at the air/water interface under surface pressures of (a) 0 and (b) 7 mN m^{-1}; (c) after annealing to 50 °C in the dry state following transfer onto a TEM grid. (ii) Enlarged image of (i).

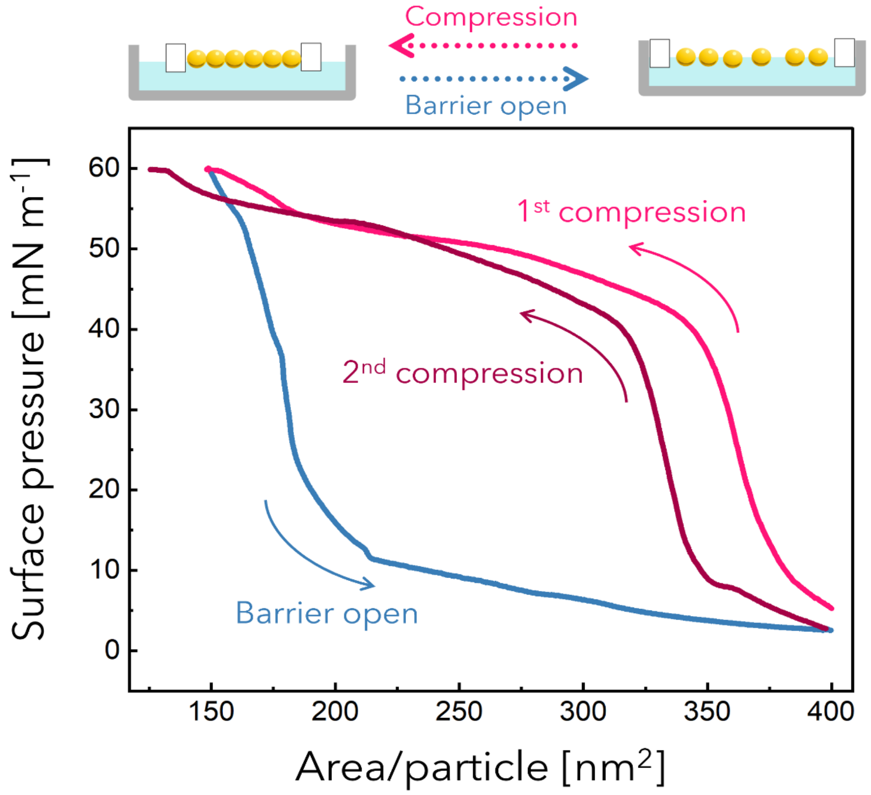

Figure S6 Pressure-area isotherm of the Au-Dend monolayer recorded over two compression and one expansion processes.

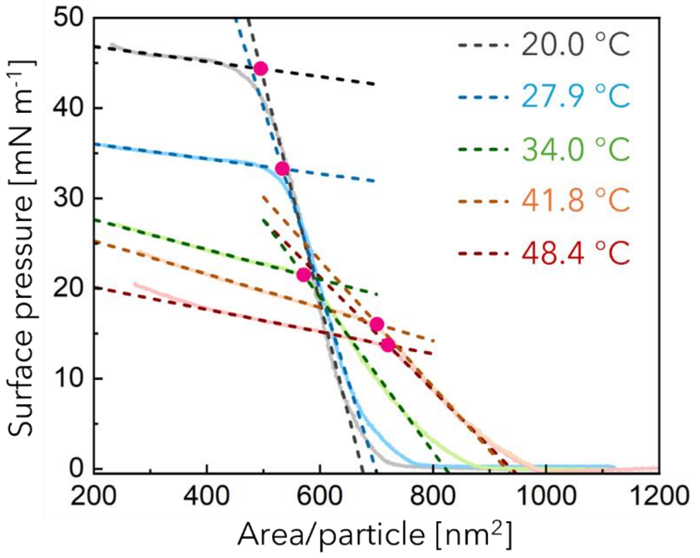

Figure S7 Definition of the transition point in π–A isotherms. The transition points represented as pink circles are determined as the surface pressure at the intersection of the tangent lines (dotted lines) drawn before and after the inflection of the curve.

In addition to the conventional definition at 10 mN m⁻¹ (open circles), three reference points were defined for determining A_{exp}: (i) 10 mN m⁻¹ (open circles), (ii) the lift-off point where the surface pressure begins to increase (open squares), and (iii) an intermediate point between the lift-off and transition points (open triangles). In Figure S8, porosity values calculated using A_{exp} obtained from each definition are plotted as a function of temperature. Table S1 summarizes the corresponding A_{exp} and porosity values for cases (i–iii).

Regardless of the A_{exp} definition employed, the Au-Dend monolayer exhibits a relatively high porosity of approximately 75% at room temperature, with a further increase of about 5% observed at elevated temperatures. These findings indicate that the Au-Dend monolayer is already loosely packed in its island-like state at room temperature, resulting in a high overall film porosity. Upon heating, the lateral arrangement evolves toward a network-like structure, accompanied by a slight increase in porosity. In other words, while the overall packing density remains largely preserved, the packing response undergoes a qualitative change.

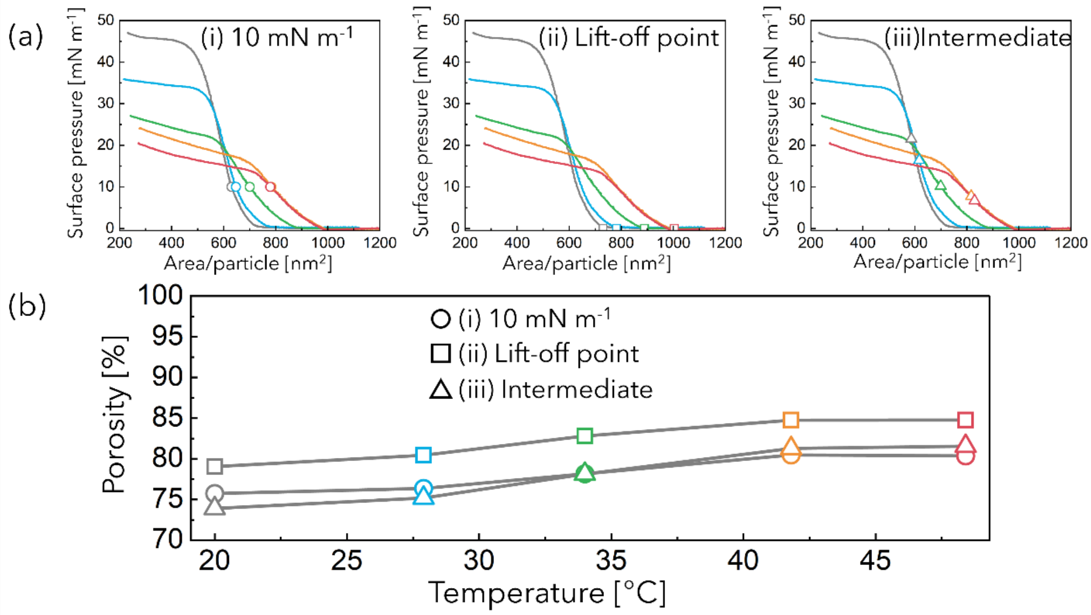

Figure S8 (a) π–A isotherms of the Au-Dend monolayer shown in Figure 3a, with selected reference points used for porosity calculations: (i) 10 mN m⁻¹ (open circles), (ii) the lift-off point (open squares), and (iii) an intermediate point between the lift-off and transition points (open triangles). (b) Porosity values calculated using A_{exp} determined according to the different definitions indicated in (a), showing a slight increasing trend with increasing temperature regardless of the A_{exp} definition.

Table S1. Summary of A_{exp} and porosity calculated using different A_{exp} determined by three different definitions shown in Figure S8.

**Temperature [°C]**	**(i) At 10 mN m^{-1}**		**(ii) The lift-off point**		**(iii) Intermediate between the lift-off and transition points**	
	A_{exp} [nm^2]	Porosity [%]	A_{exp} [nm^2]	Porosity [%]	A_{exp} [nm^2]	Porosity [%]
20.0	630.1	75.7	730.5	79.0	586.2	73.9
27.9	647.0	76.4	782.3	80.4	616.4	75.2
34.0	700.2	78.1	889.2	82.8	699.2	78.1
41.8	783.4	80.5	1002.6	84.7	816.6	81.3
48.4	779.0	80.4	1004.8	84.8	829.1	81.5

Table S2 Summary of the fitting parameters for XRR measurements of Au-Dend and Au-DT upon compression at room temperature.

sample	the surface pressure [mN/m]	the electron density of gold ρ_{Au} [e$^-$/Å^{-3}]	the radius of gold core r_{Au} [nm]	the ligand thickness d_{org} [nm]	the vertical offset of the core with respect to the water surface $\Delta_{Au/wat}$ [nm]	the vertical offset of the core in the ligand shell $\Delta_{Au/Org}$ [nm]	the electron density of the organic shell ρ_{org} [e$^-$/Å^{-3}]	the interface roughness σ [Å]	surface coverage f [-]	surface coverage of the 2nd layer f_2 [-]
Au-Dend	0	4.7	2.3	3.9	-0.40	2.0	0.38	3.1	0.25	0
	5			4.5	-0.33	2.4	0.37	2.6	0.30	
	20			5.1	-0.30	2.9	0.36	2.5	0.35	
	25			5.1	-0.33	2.9	0.34	2.5	0.35	
	30			5.9	-0.37	3.4	0.30	2.4	0.37	
Au-DT	0	4.7	3.0	0.5	8.7	0	0.25	3.1	0.63	0
	2				8.5			3.2	0.64	0.00036
	7				7.8			3.5	0.66	0.0041
	25				11.6			4.7	0.56	0.069

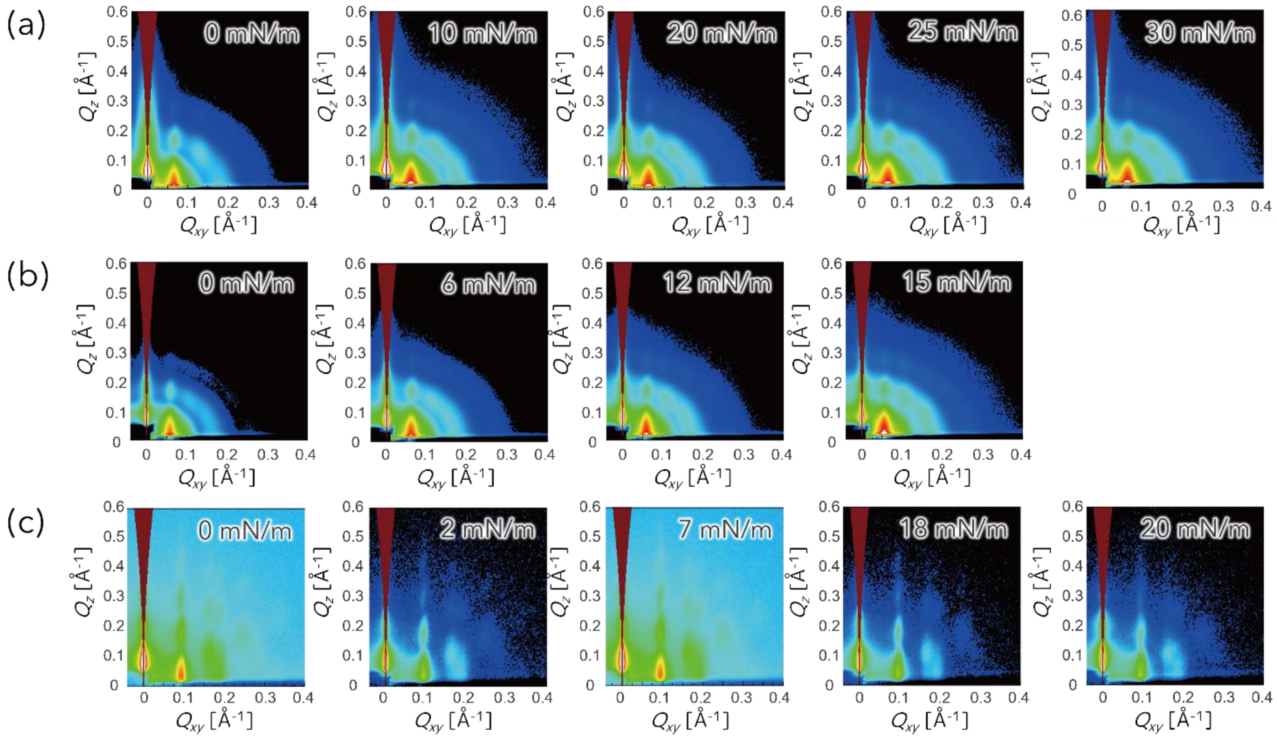

Figure S9 GISAXS images at various surface pressures of Au-Dend monolayer at (a) room temperature, (b) 40 °C, and (c) Au-DT monolayer at room temperature.

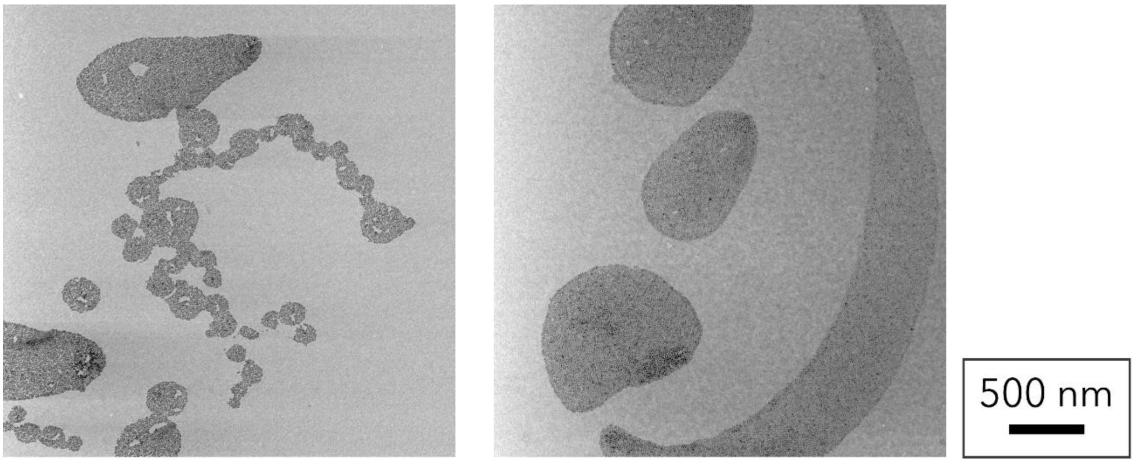

Figure S10 TEM images of Au-Dend monolayers at 27.9 °C and 0 mN/m, which suggest the coexistence of NP island and 1D chain structures.

Au-Dend was synthesized following our previously reported method[1]. In a typical procedure, both MHA and DT were introduced onto the surface of Au nanoparticles (NPs) at a molar ratio of 3:2. Subsequently, thiol-capped Au NPs and a condensing agent were dispersed in tetrahydrofuran, followed by the addition of dendron molecules to form amide bonds between the terminal amino group of the dendron and the terminal carboxyl group of MHA on the NP surface. Finally, Au-Dend was obtained by stirring the mixture for 24 h at room temperature, followed by washing five times with methanol.

Figure S11 shows Fourier transform infrared (FTIR) spectra of carboxyl-functionalized Au NPs (Au-COOH) prior to dendron functionalization, and Au-Dend. A characteristic absorption band derived from the C=O vibration was observed at 1693 cm^{-1} in the FTIR spectrum of Au-COOH. After dendron functionalization, this absorption disappeared and a new band appeared at 1657 cm^{-1}, attributable to amide bond formation, indicating that the carboxyl groups were successfully consumed to form amide linkages.

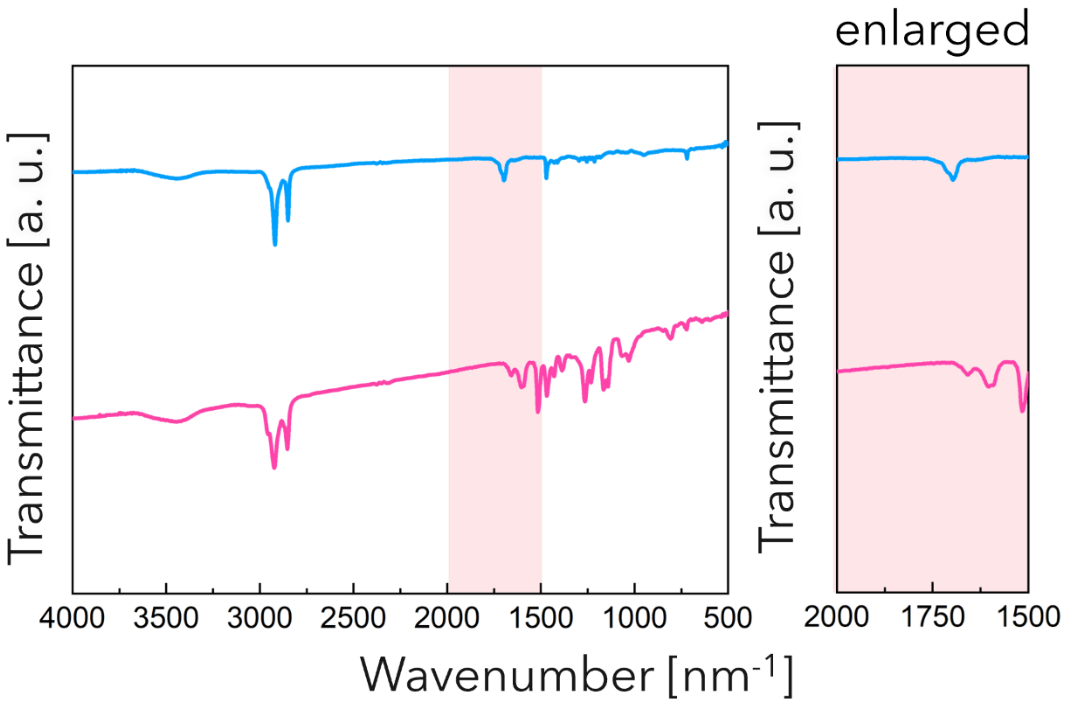

Figure S11 Fourier transform infrared spectra of Au-Dend, before (blue) and after (pink) dendron functionalization.

Thermogravimetric analysis was performed on Au-COOH and Au-Dend to determine the number of dendron molecules attached to the NP surface. As shown in Figure S12, the 19.6% difference in weight loss corresponds to the combustion of dendrons. The calculated number of dendron molecules on Au-Dend was 214 molecules per NP, with a modification density of 1.47 molecules/nm^{-2}. Reported modification densities for bulky organic ligands such as peptides or disulfide-linked ligands are

typically in the range of 1–2 molecules/nm^{-2}[5,6], supporting that Au-Dend was highly functionalized with dendron molecules, forming an Au core–organic shell structure.

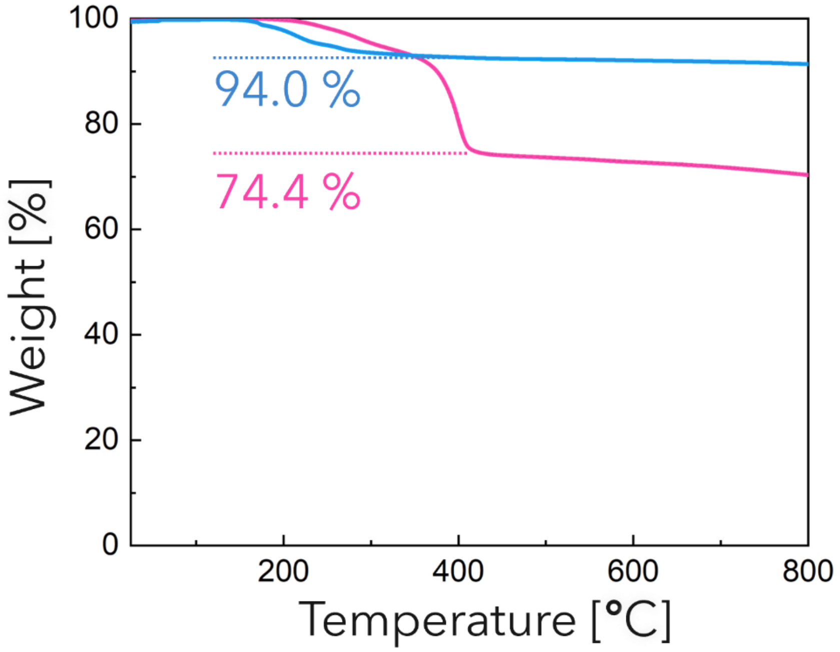

Figure S12 Thermogravimetric measurement results of Au-Dend, before (blue) and after (pink) dendron functionalization.

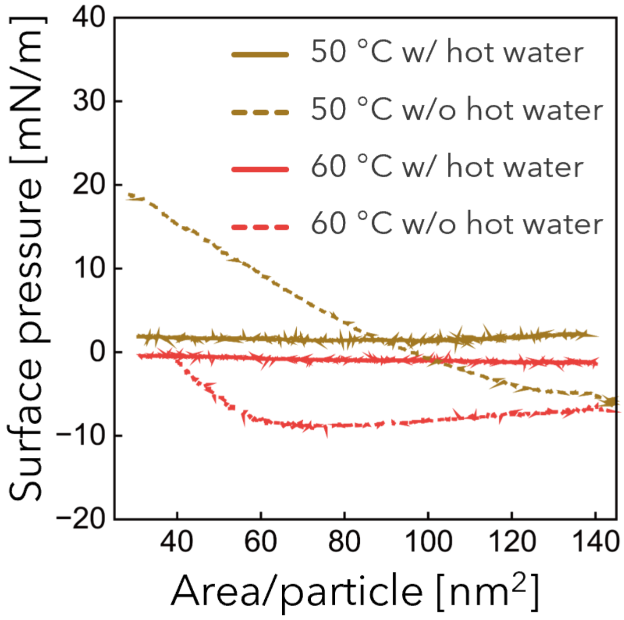

Figure S13 Effect of water evaporation on surface pressure during π–A isotherm measurements. The curves were obtained using the same trough system and experimental conditions (e.g., compression rate) as in the main experiments, but without any NP samples. The legend in the figure indicates the corresponding measurement conditions (temperature and absence of hot-water beakers).

In temperature-dependent π–A isotherm measurements, the effect of water evaporation above 50 °C had to be considered, as it can influence the measured surface pressures. As described in the Experimental section of the main manuscript, several beakers containing hot water (60 °C) were placed inside a chamber covering the trough to minimize evaporation. Figure S13 shows that, without the hot-water beakers, the surface pressure at both 50 °C and 60 °C changed significantly during compression, whereas with the beakers in place, the surface pressure remained essentially constant. Although slight fluctuations were still observed with hot-water beakers, they were ~2% relative to 30 mN/m, indicating that the effect of water evaporation was sufficiently suppressed.

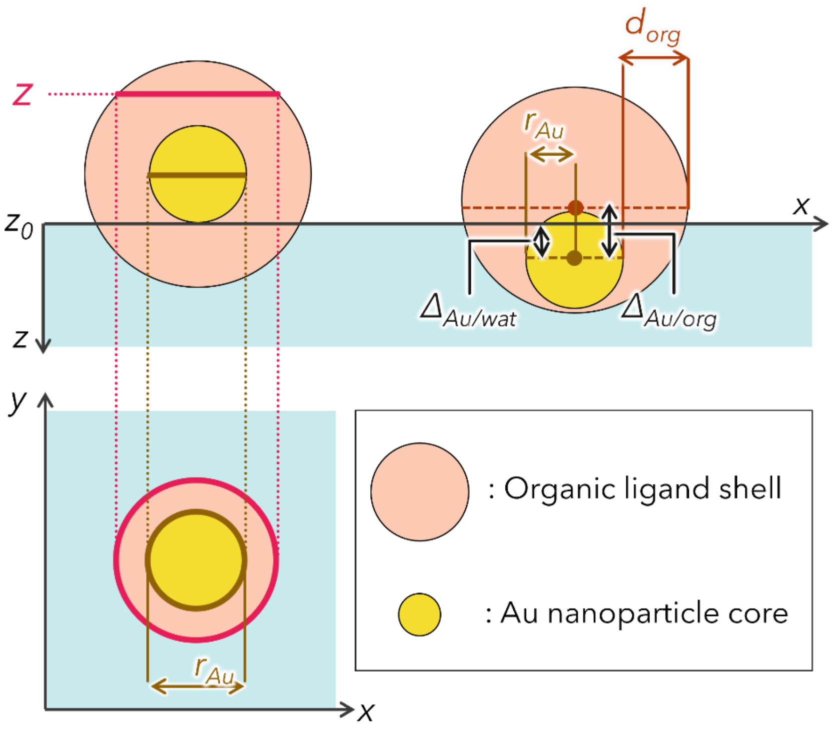

Figure S14 Schematic illustration of the fitting parameters for XRR analysis.

References

(1) Kanie, K.; Matsubara, M.; Zeng, X.; Liu, F.; Ungar, G.; Nakamura, H.; Muramatsu, A. Simple Cubic Packing of Gold Nanoparticles through Rational Design of Their Dendrimeric Corona. *J. Am. Chem. Soc.* **2012**, *134* (2), 808–811. https://doi.org/10.1021/ja2095816.

(2) Zheng, N.; Fan, J.; Stucky, G. D. One-Step One-Phase Synthesis of Monodisperse Noble-Metallic Nanoparticles and Their Colloidal Crystals. *J. Am. Chem. Soc.* **2006**, *128* (20), 6550–6551. https://doi.org/10.1021/ja0604717.

(3) Pusterla, J.; Scoppola, E.; Appel, C.; Mukhina, T.; Shen, C.; Brezesinski, G.; Schneck, E. Characterization of Lipid Bilayers Adsorbed to Functionalized Air/Water Interfaces. *Nanoscale* **2022**, *14* (40), 15048–15059. https://doi.org/10.1039/D2NR03334H.

(4) Mortara, L.; Mukhina, T.; Chaimovich, H.; Brezesinski, G.; van der Vegt, N. F. A.; Schneck, E. Anion Competition at Positively Charged Surfactant Monolayers. *Langmuir* **2024**, *40* (13), 6949–6961. https://doi.org/10.1021/acs.langmuir.3c04003.

(5) R. Ivanov, M.; J. Haes, A. Anionic Functionalized Gold Nanoparticle Continuous Full Filling Separations: Importance of Sample Concentration. *Anal. Chem.* **2012**, *84* (3), 1320–1326. https://doi.org/10.1021/ac2022376.

(6) Li, J.; Chen, Y.; Kawazoe, N.; Chen, G. Ligand Density-Dependent Influence of Arginine–

Glycine–Aspartate Functionalized Gold Nanoparticles on Osteogenic and Adipogenic Differentiation of Mesenchymal Stem Cells. *Nano Res.* **2018**, *11* (3), 1247–1261. https://doi.org/10.1007/s12274-017-1738-5.
